# Using Clustering to Examine Inter-Individual Variability in Topography of Auditory Event-Related Potentials in Autism and Typical Development

**DOI:** 10.1007/s10548-021-00863-z

**Published:** 2021-07-22

**Authors:** Patrick Dwyer, Xiaodong Wang, Rosanna De Meo-Monteil, Fushing Hsieh, Clifford D. Saron, Susan M. Rivera

**Affiliations:** Department of Psychology, UC Davis, Center for Mind and Brain, UC Davis; Department of Statistics, UC Davis; Center for Mind and Brain, UC Davis; Department of Statistics, UC Davis; Center for Mind and Brain, UC Davis, MIND Institute, UC Davis; Department of Psychology, UC Davis, Center for Mind and Brain, UC Davis, MIND Institute, UC Davis

**Keywords:** Autism, heterogeneity, clustering, event-related potentials (ERPs), sensory processing

## Abstract

**Objective:**

Although prior studies have compared sensory ERP responses between groups of autistic and typically-developing participants, it is unclear how heterogeneity contributes to the results of these studies. The present study used examined individual differences in these responses.

**Method:**

130 autistic children and 81 typically-developing children, aged between 2–5 years, listened to tones at four identity levels while 61-channel electroencephalography was recorded. Hierarchical clustering was used to group participants based on rescaled event-related potential (ERP) topographies between 51–350ms.

**Results:**

The hierarchical clustering analysis revealed substantial heterogeneity. Some of the seven clusters defined in this analysis were characterized by prolonged fronto-central positivities and/or weak or absent N2 negativities. However, many other participants fell into clusters in which N2 responses were present at varying latencies.

**Conclusions:**

Atypical response morphologies such as absent N2 responses and/or prolonged positive-going responses found in some autistic participants may account for prior research findings of attenuated N2 amplitudes in autism. However, there was also considerable overlap between groups, with participants of both groups appearing in all clusters.

**Significance:**

These results emphasize the utility of using clustering to explore individual differences in brain responses, which can expand on and clarify the results of analyses of group mean differences.

## Introduction

Autism spectrum development (ASD)^1^ is a heterogeneous neurominority. It is diagnosed behaviourally, and in contemporary guidelines, diagnosis is based on the presence of atypical social and communication behaviours, as well as at least some atypical non-social behaviours, such as motor stereotypes (“stimming”), intensely focused interests, and/or atypical sensory reactivity (American Psychiatric Association, 2013). Although autism has traditionally been described using a spectrum metaphor, implying a single continuous dimension of greater to lesser severity, a more recent view describes autism as a constellation varying along multiple dimensions (Fletcher-Watson & Happé, 2019). The diversity within autism includes cognitive and behavioural heterogeneity along different dimensions, such as social-communication characteristics of autism (Georgiades et al., 2013); different dimensions of non-social autism characteristics such as intense interests and insistence on sameness (Grove, Begeer, Scheeren, Weiland, & Hoekstra, 2021); cognitive ability and different dimensions thereof such as verbal comprehension and fluid reasoning (Audras-Torrent et al., 2021); different forms of sensory sensitivity such as hyperacusis and misophonia (Williams, He, Cascio, & Woynaroski, 2020b); and the presence and severity of co-occurring conditions such as anxiety and depression (McCauley, Elias, & Lord, 2020); to name only a few. In general, autistic individuals differ from one another along at least as many distinct dimensions as typically-developing people. Moreover, there appears to be substantial neural heterogeneity in autism. For example, combining across research sites, functional connectivity appears to have only a relatively limited capacity to diagnostically separate autistic individuals from typically-developing controls (Lanka et al., 2019). Moreover, such binary diagnostic classification seems less demanding than attempting to discriminate between typical development (TD), autism, and other atypical neurotypes, such as attention-difference hyperactivity development (ADHD),^2^ let alone combinations of co-occurring atypical neurotypes. In this context, it hardly seems surprising that some scholars in the social sciences emphasize socially constructed aspects of ASD, rather than the neurotype’s biological coherence (Grinker, Yeargin-Allsopp, & Boyle, 2011).

This inter-individual variability may contain important information. For example, sensory processing in autism appears to be an area of profound real-world significance, being related not only to social cognition (Green, Hernandez, Bowman, Bookheimer, & Dapretto 2018), but also to quality of life (Lin & Huang, 2019) and anxiety (Green, Ben-Sasson, Soto, & Carter, 2012; Neil, Olsson, & Pellicano, 2016). Autistic sensory processing is also highly heterogeneous (Uljarević et al., 2017). It therefore seems eminently reasonable to expect that meaningful inter-individual variability exists in sensory-related brain responses in autism. For example, in a sample of 2-, 3-, and 4-year-old autistic and typically-developing children, Dwyer et al. (2020) examined neural heterogeneity by using clustering to group participants based on the relative strengths of topography-independent responses to sounds of different intensities. Substantial variability existed in both diagnostic groups, and notably, in ASD, relatively stronger responses to high-intensity sounds were related to caregiver-reported auditory distractibility/filtering problems. The present study extends the exploration of neural heterogeneity in the same sample studied by Dwyer et al (2020) with a focus on the spatiotemporal heterogeneity in electrocortical responses not captured in our initial examination of these data using Global Field Power (GFP).

Canonically speaking, young children in the 2–5 year age range of the present study exhibit two principal auditory ERP components over fronto-central channels, the P1 and N2 (Čeponienė, Lepistö, Alku, Aro, & Näätänen, 2003; Shafer, Yu & Wagner, 2015), as well as a large temporal negativity (Bruneau, Roux, Adrien, & Barthélémy, 1997; Shafer et al., 2015). This temporal negativity is variously referred to as Tb (Ponton, Eggermont, Khosla, Kwong, & Don, 2002), Na (Shafer et al., 2015), or N1_c_ (Bruneau et al., 1997); the present study refers to this response as the “Tb.” Additional temporal responses have been described, but these are not consistently observed in the age range of the present study (Shafer et al., 2015). The fronto-central auditory N1 and P2 responses commonly associated with auditory ERPs are not typically apparent until later in development, around ages 9–14 (Albrecht, Suchodoletz, & Uwer, 2000; Gilley, Sharma, Dorman, & Martin, 2005; Ponton et al., 2002; Sharma, Kraus, McGee, & Nicol, 1997; cf. Wunderlich, Cone-Wesson, & Shepherd, 2006). There are also developmental changes in topography of auditory ERPs. While the P1 is observed fronto-centrally in young children such as those in the present study, the P1 and the later-emerging N1 shift to a more central topography by adulthood (Čeponienė, Rinne, & Näätänen, 2002). Source localization suggests the location of the equivalent cortical dipoles generating these responses are fairly similar, though delayed in children compared to adults, with origins in temporal auditory cortex (Albrecht et al., 2000, Parviainen, Helenius & Salmelin, 2019; Ponton et al, 2002). Although developmental ERP studies sometimes report the proportion of individuals classified as displaying vs. not displaying a particular ERP component (e.g., Gilley et al., 2005; Shafer et al., 2015), more systematic examination of heterogeneity could uncover different subgroups of individuals with particular ERP morphologies and topographies. Observed scalp ERP waveforms are the consequence of the superposition of numerous synchronized cortical neurons (typically pyramidal cells) active as arrays of effective dipoles, which, as a function of dipole orientation and polarity related to folding of the cortical mantle, may cancel each other out (Luck, 2014). Individuals could differ in the relative strengths or contributions of these numerous active cortical effective dipoles to their observed ERP responses. Maturational changes, such as degree of myelination, could influence differences in the relative strength or importance of different regional cell populations contributing to observed scalp surface recordings. The orientation of dipoles could also vary, especially as brain structures continue to develop. In addition, prior research indicates that positions of EEG electrodes, relative to neuroanatomical features, vary across individuals (Homan, Herman, & Purdy, 1987). This would be expected to contribute additional variability in observed topographies beyond individual differences in patterns of cortical activation to a given stimulus.

Inter-individual variability due to such factors could complicate the interpretation of conventional ERP statistical analyses. For example, a finding that a particular ERP component has significantly lower amplitude in a particular group or condition could reflect any of a number of possibilities: a major dipole contributing to said component might be slightly weaker in all participants, said dipole might be substantially weaker in some participants but largely intact in others, another dipole with opposing polarity could be stronger, etc. The statistical analysis, however, would not distinguish between these possibilities. Descriptive techniques, such as using data-driven clustering to sort participants into subgroups that could illustrate different individual ERP patterns underlying grand-averaged patterns, could therefore provide additional information beyond that offered by conventional analyses.

The investigation of inter-individual variability might also help clarify inconsistencies in prior research regarding canonical ERP amplitudes in ASD. In some studies, no differences in amplitudes of auditory responses such as the P2 and N2 are observed between autistic and typically-developing groups (e.g., Andersson, Posserud, & Lundervold, 2013; Salmond, Vargha-Khadem, Gadian, de Haan, & Baldeweg, 2007). However, while Orekhova et al. (2008) found no evidence of group differences in P1 amplitudes to the first sound in pairs of clicks, Orekhova et al. (2009) found that amplitudes of other responses to these initial clicks were weaker in autistic participants: specifically, Tb amplitudes were lower over the right hemisphere and N2 amplitudes were weaker frontally. Other studies find that autistic participants have, relative to typically-developing participants, as well as non-autistic individuals with intellectual disabilities, bilaterally weaker Tb responses to pure tones across a range of sound intensities (Bruneau et al., 1999; Bruneau, Bonnet-Brilhault, Gomot, Adrien, & Barthélémy, 2003). Weaker N2 responses have also been observed in other studies (Donkers et al., 2020; Yu et al., 2018). Whitehouse and Bishop (2008) observed normal P1 and N2 amplitudes to nonspeech sounds (complex tones) in autism, but (at a trend level) diminished P1 and (significantly) diminished N2 amplitudes to speech sounds (vowels). While some of the inconsistency in these findings appears to reflect differences in experimental procedures and stimuli, it also seems likely that the heterogeneity of the autistic population could contribute to variability in results. There is also variability in findings related to ERP and event-related field latencies; although a number of studies have observed delayed auditory responses in ASD generally (Bruneau et al., 1999; Matsuzaki et al., 2019; Port et al., 2016; Roberts et al., 2010), specific subgroups might show particularly delayed responses (Roberts et al., 2019).

If different latent populations within the autistic constellation exhibit different patterns of neural responses, different studies might obtain different results depending on which aspects of population variability they most heavily, but unknowingly, tap into, based on factors such as inclusion criteria and recruitment sources. With rare exceptions (e.g., DiStefano, Senturk, & Jeste, 2019; Dwyer et al., 2020), clustering analyses are not generally employed to investigate heterogeneity of electrophysiological responses in autism. This is unfortunate, as information from such analyses could potentially help researchers contextualize and better understand results at the level of group means.

The present study aims to use clustering to explore different patterns of auditory ERPs that may be seen within the autistic constellation, in addition to comparing autistic and typically-developing groups in a search for mean differences. To provide sufficient participants for this clustering analysis, a large sample of young autistic and typically-developing children was recruited as part of the Autism Phenome Project (APP) at the UC Davis MIND Institute. ERPs in this sample were recorded in response to sounds of different intensities (50, 60, 70, and 80 dB). This dataset has previously been used to cluster children based on the relative strengths of topography-independent responses (Global Field Power, GFP) from an early time window approximately corresponding to that of the P1 response (Dwyer et al., 2020); the present study extends this work by not only including responses in sliding time intervals across an expanded time window, but also by including information about the topography of responses over different scalp regions.

It is important to note that the present study’s use of clustering is not meant to imply that the subgroups defined here exist as discrete categorical entities. The question asked by algorithms aiming to determine an optimal number of clusters is ill-posed (Fushing & McAssey, 2010), at least when clusters are not convex and well-separated. When clusters are poorly separated and non-convex, this may imply that the structure of the data is in fact dimensional rather than categorical, in which case the idea of an optimal or true number of clusters becomes problematic. (Conversely, when clusters are clearly convex, this may be visually obvious, rendering clustering analyses potentially superfluous.) Thus, we view the use of clustering procedures as a descriptive data exploration technique that can complement conventional ERP analyses. We are not claiming that the particular clusters revealed here are inherent in any population of similarly distributed individuals. Rather, we aim to parse interindividual variability of electrocortical responses in *this* dataset into bounded subgroups that would ordinarily be lumped together. Thus, imposing categories on dimensional data may nonetheless yield valuable descriptive information, and different clustering solutions with varying numbers of categories at different hierarchical levels may offer valid descriptions of the same data for different purposes. We have also chosen to cluster autistic and typically-developing participants together, so that results in each diagnostic group can be placed in context through comparison to the other group.

Although the use of clustering in the present study should be regarded as exploratory, prior research reporting that Tb and N2 amplitudes are attenuated in ASD (reviewed by Williams, Abdelmessih, Key, & Woynaroski, 2020a) could suggest that atypical response morphologies in time windows and over scalp regions canonically associated with these ERP components will be observed in some subgroups within ASD, while other subgroups will show canonically-expected patterns. Indeed, prior research with the present dataset suggests that amplitudes over the spatiotemporal window of the N2 are indeed attenuated in ASD (Dwyer et al., 2021). As such, we predict:

1. Some clusters containing a disproportionate number of autistic participants will be characterized by atypical topographies and weaker/less negative N2 responses, although other autistic participants will be placed with predominantly typically-developing participants in clusters with more typical topographies and robust N2 responses.
2. Some clusters will show evidence of ERP responses with morphologies and topographies that differ from those canonically described in prior research with children in this age range, such that participants’ responses cannot be easily described in terms of these canonical patterns.

## Materials and Methods

### Participants

As part of the APP, attempts were made to collect ERP data from 243 autistic and 96 typically-developing children, aged between 2–5. Autistic participants were required to meet criteria for a pervasive developmental disorder (based on DSM-IV and Collaborative Programs of Excellence in Autism Network criteria) and reach ADOS-G (Lord et al., 2000) autism spectrum cut-off scores as well as cut-offs for either the social or communication subscales of the ADI-R (Lord, Rutter, & Le Couteur, 1994). Further information about the APP and participant recruitment can be found in previous publications (e.g., Libero et al., 2016; Nordahl et al., 2011; De Meo-Monteil, 2019; Dwyer et al, 2020). A number of participants were excluded from the present study due to failure to collect data, due to noisy data, due to an insufficient number of acceptable-quality trials (<400), due to an excessive number of poor-quality channels (>6–7), or due to the presence of neuroanatomical abnormalities revealed by magnetic resonance imaging collected in the APP. One participant entered the study in the typically-developing group but was diagnosed with autism at a later APP time-point; this participant’s data are also excluded. The final sample of children with usable electrophysiological data includes 81 typically-developing participants (52 male) and 130 autistic participants (110 male) (Table 1). Families received a gift card in return for their participation in the study. The study was approved by the UC Davis Institutional Review Board and informed consent was obtained from the parent/guardian of each participant.

### Measures

Cognitive ability was measured with the Mullen Scales of Early Learning (MSEL; Mullen, 1995). In the APP, four MSEL subscales were administered: Visual Reception (VR), Fine Motor (FM), Expressive Language (EL), and Receptive Language (RL). A ratio developmental quotient (DQ) was calculated by dividing mental age by chronological age, then multiplying by 100. MSEL data are available from all 130 autistic participants with usable electrophysiological data, and for 80 of the 81 typically-developing participants.

Adaptive functioning was assessed with the parent-report form of the Vineland Adaptive Behavior Scales, Second Edition (VABS-II; Sparrow, Cichetti, & Balla, 2005). The standardized composite adaptive behaviour score was used to index individuals’ overall adaptive functioning for analyses. VABS scores were available from 105 autistic (97 male) and 69 typically-developing participants (43 male).

The Short Sensory Profile (SSP; McIntosh, Miller, & Shyu, 1999) was collected as a measure of caregiver-reported sensory behaviours. Higher scores reflect more typical sensory behaviours, while lower scores reflect atypical sensory behaviours that may be problematic. Although the original seven SSP subscales were defined in a typically-developing sample (McIntosh et al., 1999), two studies have investigated SSP factors in samples of autistic children (Tomchek, Huebner, & Dunn, 2014; Williams, Failla, Gotham, Woynaroski, & Cascio, 2018). The present study employs the more recent nine-factor solution developed by Williams et al. Specifically, the present study examines SSP total scores (calculated on the basis of all 38 items) and the three factor scores that appear to tap into the auditory modality, namely: Auditory Distractibility, Hyporesponsiveness to Speech, and Noise Distress. Complete SSP data were available from 98 autistic (81 male, M_Age_ = 38.87 months) and 65 TD participants (42 male, M_Age_ = 37.28 months). Partial data that included some or all of the factors of interest were available from another nine participants.

### EEG Task

Participants were seated on a caregiver’s lap in a dimly-lit, audiometrically-quiet, shielded chamber. Stimuli were 50ms (including 5ms rise and decay time) complex tones, each consisting of sine waves of equal amplitude overlaid at the following 7 frequencies (musical notes): 249 Hz (B3); 616 Hz (D5), 788 Hz (G5), 1042 Hz (C6), 1410 Hz (F6), 1952 Hz (B6), and 2749 Hz (F7). All tones were identical to one another in terms of the proportion of stimulus energy drawn from each frequency, and thus were identical in terms of frequency spectra. However, tones randomly varied in intensity (50 dB, 60 dB, 70 dB, and 80 dB SPL); tones of the same intensity were never presented twice in succession. Tones were presented at a randomly variable ISI of 1–2s using Sony MDR-222KD headphones calibrated with a B&K artificial ear (model 4153) and sound meter (model 2229). While they passively listened to these tones, participants watched a quiet video of their or their caregiver’s choice. Approximately 1200 trials (~300 trials/condition) were collected from each participant, with breaks included when required. Further details regarding the experimental setup are available in De Meo-Monteil et al. (2019).

### EEG Data Acquisition and Processing

EEG was collected with a 61-channel cap (www.easycap.de) and a Compumedics Neuroscan Synamp II amplifier. Data were sampled at a rate of 1000Hz with Cz as a reference. Data were then average-referenced and filtered offline with a low cut-off of 0.4 Hz (12dB/octave roll-off). Given the study’s goal of exploring individual differences in ERP data, we sought to maximize the event-related signal-to-noise ratio by removing putatively non-neural signal sources from the data using an intensive data processing pipeline. Epochs (spanning −200ms to 900ms, including 300ms necessary for subsequent independent components analysis) were screened and extreme amplitudes removed using the artifact scan tool of BESA 5.2 (www.besa.de), amplitude thresholds were adjusted manually to optimize retention of usable data and rejection of extreme artefacts (e.g., temporary channel disconnection, gross movements). Mean amplitude thresholds were 316.76 μV (*SD* = 98.74) in ASD and 303.81 μV (*SD* = 95.09) in TD. Data were then manually inspected and clear artefacts (e.g., temporary channel disconnections) not removed by the amplitude threshold were rejected manually. On average, in the ASD group, 23% of trials were removed in this process, compared to 19% in the TD group (see also Table 2). Remaining epochs were submitted to a Second-Order Blind source Identification (SOBI; Belouchrani, Abed-Meraim, Cardoso, & Moulines, 1997; Tang, Sutherland, & McKinny, 2005) independent components analysis. A semi-automatic artifact removal tool (SMART, https://stanford.edu/~saggar/Software.html) was used to identify signal sources from SOBI that were manually interpreted, on the basis of outputs depicting signal source topography, spectra, autocorrelation, and time series, to be of non-neural origin (such as EMG, EOG, and blinks). This SOBI analysis was performed separately on the first and second halves of the data from each participant, consistent with recommendations (Luck, 2014). Additional details regarding artifact removal using SOBI and SMART are discussed in Saggar et al. (2012). Artifact-free trials were then reconstructed from the putatively neural SOBI signal sources and inspected to verify a lack of noise; putative noise reconstructions were also created to verify absence of neural signal therein. Finally, separate averages for each of the four intensity conditions were computed for each subject. Data from excluded channels were interpolated using a spherical spline (Perrin, Pernier, Bertrand, Giard, & Echallier, 1987). Epochs (now spanning 100ms pre-stimulus onset to 600ms post-stimulus onset) were filtered (second-order Butterworth with −12dB/octave roll/off; 40Hz low-pass; 60Hz notch) and baseline-corrected using the pre-stimulus period with Cartool software (Brunet et al., 2011).

### EEG Data Analysis

In order to focus on inter-individual differences in neural responses, and given that the absolute strength of observed ERPs can be influenced by non-neural biophysical factors such as skull thickness (Frodl et al, 2001), we chose to rescale the data to emphasize differences in response strength between intensity conditions, as well as response topography. Therefore, separately at each time-point, each participant’s data were rescaled such that the individual participant’s highest ERP amplitude in any condition or channel at that time point became 1, while the lowest (most negative) amplitude became 0.

**Fig. 1.**
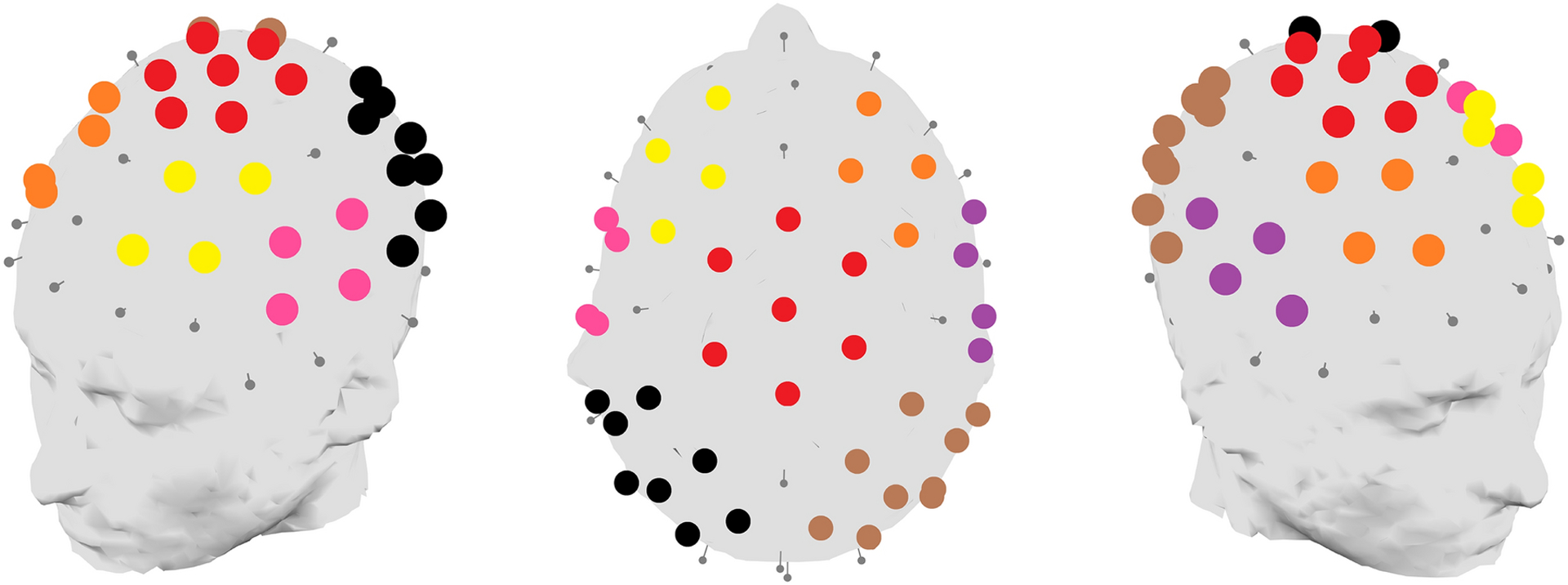
Seven regions of interest over the scalp were defined for the purposes of the topographic clustering analysis; each is indicated on these three-dimensional head plots through a separate colour. The seven scalp regions are: Central (red); Left Frontal (yellow); Right Frontal (orange); Left Temporal (pink); Right Temporal (violet); Left Posterior (black); Right Posterior (brown).

Seven regions of interest across the scalp were defined: a central region, left and right frontal regions, left and right temporal regions, and left and right posterior regions (Fig. 1). These regions were selected based on visual inspection of grand-average topographies: the central and frontal regions aimed to capture the P1 and N2 responses while the temporal regions aimed to capture the Tb response. Each participant’s rescaled ERP responses were averaged across electrodes within the seven regions of interest in consecutive 25ms time-windows between 51 – 350ms; these data were then submitted to the hierarchical clustering analysis. Data from electrodes lying on the edge of the cap, as well as certain channels lying between regions, were discarded.

In that analysis, we used Ward’s method to hierarchically cluster participants based on the topography and strength of their auditory electrophysiological responses. Ward’s method describes clusters in multivariate Euclidean space by successively adding clusters together in order to minimize their variance. This hierarchical process begins with clusters representing a single participant, but clusters grow as they are combined with further clusters until the entire dataset is contained within a single cluster. The process generates a dendrogram depicting the clusters that exist at different hierarchical levels.

The number of clusters were determined based on Euclidean distances between clusters (reflected in the height of the dendrogram branches), the interpretability and meaning of the clusters, and the stability of the results when subsamples of participants were repeatedly extracted and re-clustered (see Appendix B).

To further describe and interpret the ERPs found in the clusters, we also used traditional component-based analyses to statistically compare ERPs across clusters. These results are presented in Appendix C.

Exploratory Kruskal-Wallis and Wilcoxon-Mann-Whitney tests were used to compare clusters on chronological age, MSEL DQ, VABS composite scores, and SSP total, auditory distractibility, hyporesponsiveness to speech, and noise distress scores.

## Results

**Fig. 2.**
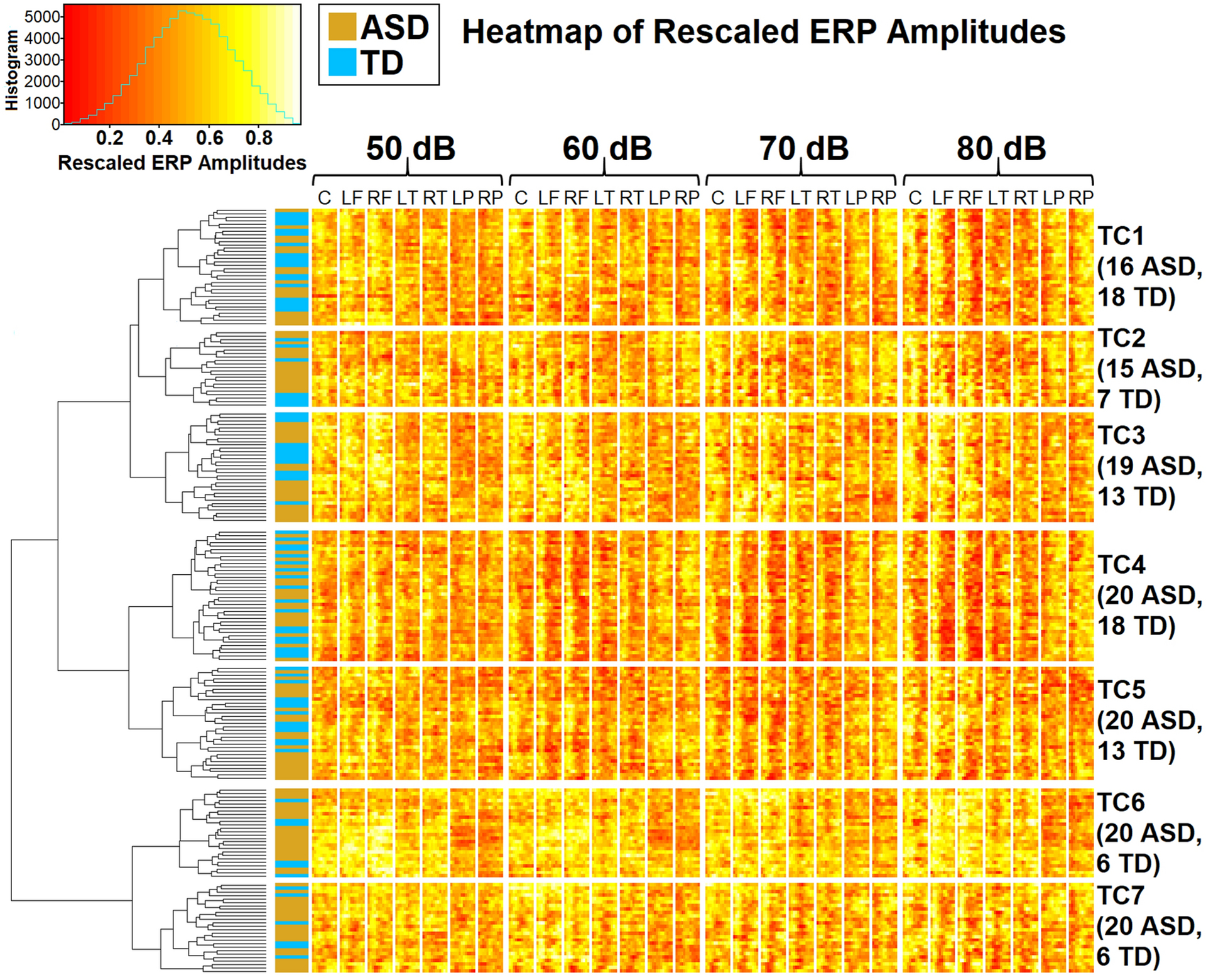
ERP amplitudes, rescaled within participants and time-points to range between 0 and 1 across conditions and channels, as clustered using Ward’s method. The vertical axis shows participants (gold indicates autistic, blue indicates typically-developing); lines on this axis form a hierarchical dendrogram showing clusters at different levels, with branch height indicating Euclidean distances between clusters. The horizontal axis depicts the four intensity conditions and seven pre-defined scalp regions (C: Central; LF: Left Frontal; RF: Right Frontal; LT: Left Temporal; RT: Right Temporal; LP: Left Posterior; RP: Right Posterior). Within each scalp region and condition, consecutive 25ms windows between 51 and 350 ms are shown from left to right. Separate clusters and scalp regions on the vertical and horizontal axes are divided by blank white space. The scale is provided by a histogram in the upper left corner; the horizontal axis of the histogram shows rescaled amplitudes. Brighter (more yellow/white) colours reflect higher rescaled amplitudes, while darker (more red) colours reflect lower rescaled amplitudes. Topographic Clusters 1TC1through 7 (TC1–7) are labelled within the dendrogram and on the right, where Ns are provided by group and cluster.

**Fig. 3.**
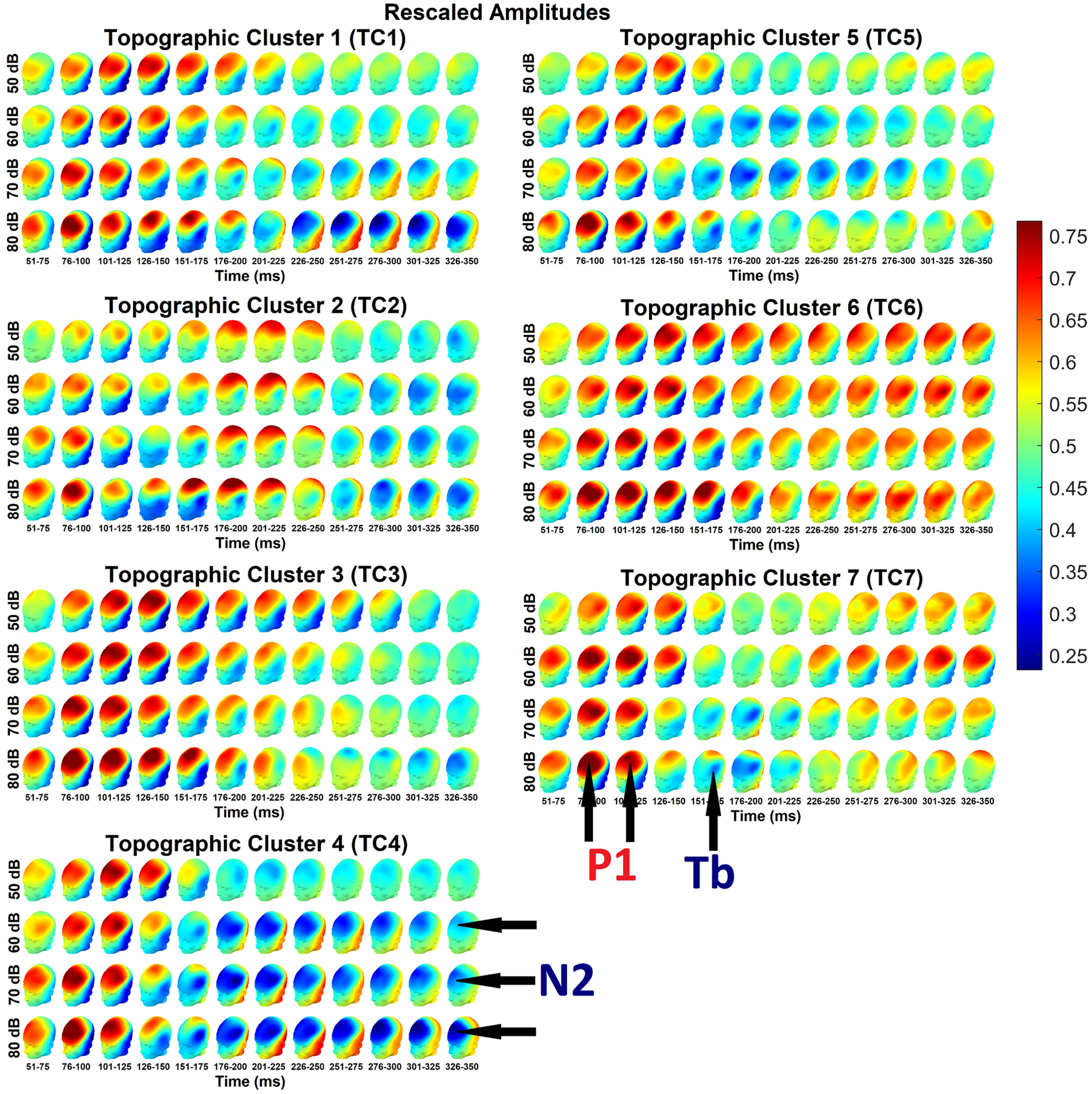
ERP amplitudes, rescaled within participants and time-points to range between 0 and 1 across conditions and channels, averaged across each cluster and intensity, collapsed across diagnostic groups, spherically splined from 61 channels and plotted on a head model, in consecutive 25 ms time windows from 51– 350 ms. All clusters appear to display frontocentral P1 responses around ~76–175 ms, though with lower amplitude in TC2. Interestingly, in TC6, the frontal P1 response appears extremely prolonged, with high rescaled voltages being visible across all time windows and intensities from ~76–350 ms. TC7 also displays late high frontocentral rescaled amplitudes, but these only become visible after ~225 ms and are less visible in the 80 dB condition. In TC2, the P1 appears to be followed by a second period of high rescaled amplitudes over central sites around ~176–250 ms (i.e., a central positivity, as demonstrated by raw amplitudes topographies in Fig. 4); a prolongation of the P1 can be observed in TC3 up to as late as ~251–275 ms. Frontocentral N2 responses are visible in TC1, TC2, TC3, TC4, and TC5, and to some extent TC3, but onset latencies vary substantially from around ~176–200 ms (TC4) to ~301–325 ms (TC3). Some suggestion of an N2 is visible in TC7 (~176–200 ms), though this might be better interpreted as a Tb response. The N2 is generally evoked mainly in response to high-intensity 70 dB and 80 dB sounds, but clear N2 responses to soft 60 dB sounds are visible in TC2, TC4, and TC5. Strangely, there is little evidence of an N2 response to high-intensity 80 dB sounds in TC5.

For the purposes of the present analysis, we decided a seven-cluster solution appeared to offer the best description of the data (Fig. 2; see also raw voltage heatmap in Supplementary Fig. A.1). We designated these clusters Topographic Clusters 1–7 (TC1-TC7). The rescaled topography patterns for each cluster (depicted in separate sections of the figure, see caption for details), intensity (represented by rows), and consecutive 25 ms averaged time windows (represented by columns) are also displayed more accessibly in Fig. 3; these patterns will be described more completely later in the results section.

### Cluster Membership by Diagnostic Group and Sex.

Proportions of participants in each diagnostic group did not significantly vary across clusters in the seven-group solution, *X*^2^ (6, N = 211) = 9.98, *p* = .13 (Table 3). Given visually-apparent trends for there to be relatively few typically-developing participants in clusters such as TC6 and TC7 (see the left column of Fig. 2; autistic participants are represented in gold and typically-developing participants in blue), this may reflect the fairly low statistical power of the chi-square statistic. There was no evidence suggesting cluster membership varied by sex in either autistic, *X*^2^ (6, N = 130) = 0.97, *p* = .99, or typically-developing, *X*^2^ (6, N = 81) = 7.85, *p* = .25 individuals (Table 4).

### P1 Topographies.

In Fig. 3, the P1 response can be seen fronto-centrally in head plots from time window columns towards the left of each cluster (particularly the second to fourth from the left, around ~76 – 150 ms; see also raw voltage topographies in Supplementary Fig. A.2).

Generally speaking, the topographies of the P1 response were similar across clusters, and, at least for the 76–100 ms (2^nd^) column, show evidence of intensity-dependence. The P1 response appeared markedly weaker in TC2 than in other clusters, especially over medial channels. In addition, in TC5, responses to 50 dB, 60 dB, and 70 dB sounds (the top three intensity rows in Fig. 3) appeared relatively weaker in comparison to the 80 dB response (from the bottom intensity row) than in other clusters.

### Later Positivity Topographies

**Fig. 4.**
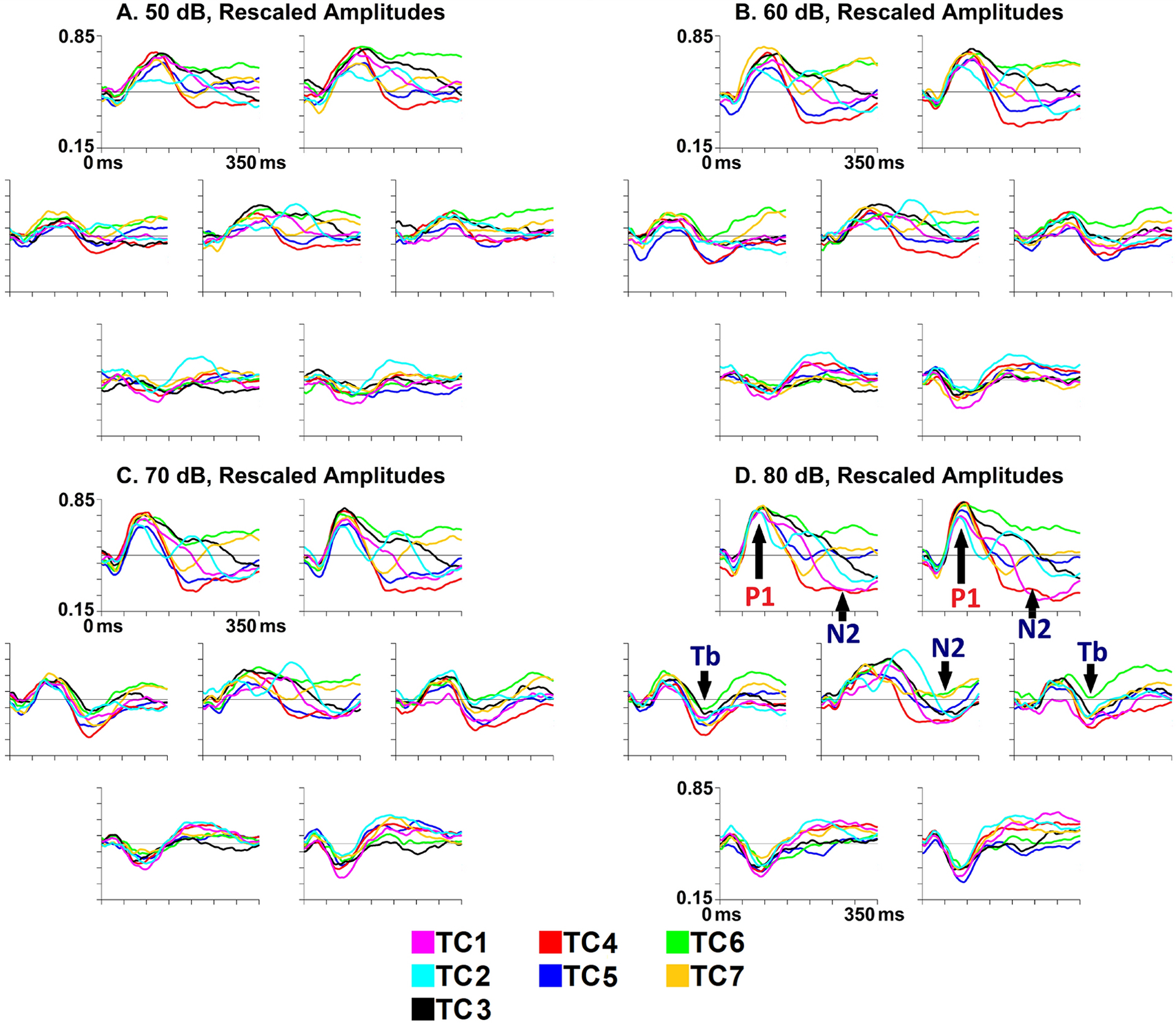
ERP amplitude waveforms over electrodes in each of the seven regions depicted in Fig. 1, rescaled within participants and time-points to range between 0 and 1 across conditions and channels, averaged across each cluster from the seven-cluster solution, collapsed across diagnostic groups. The Y-axis (vertical line on each subplot) ranges from 0.15 to 0.85, with tick marks for every 0.10 rescaled amplitude units. The X-axis ranges from 0 to 350 ms, with tick marks each 50 ms. *A* (top left). Rescaled amplitude waveforms evoked by 50 dB stimuli. All clusters appear to display frontocentral P1 responses. Interestingly, TC6 displays continuous high rescaled amplitudes (suggestive of positive raw voltages) frontally, perhaps suggestive of a very prolonged P1 response. In TC2, the P1 is followed by a second period of high rescaled amplitudes (suggestive of a positivity) over the central region, and to a lesser extent frontally. The central P1 appears somewhat prolonged in TC1 and TC3. It is difficult to discern any clear N2 response in this condition, but frontocentral rescaled amplitudes appear particularly low in TC4. *B* (top right). Rescaled amplitude waveforms evoked by 60 dB stimuli. In TC7, a period of low rescaled frontal amplitudes (i.e., a negativity, perhaps an early N2) following the P1 terminates early and appears to be followed by a frontocentral positivity. As in the 50 dB condition, a prolonged frontal positivity is visible in TC6. A second positivity following P1 appears evident in TC2, while the P1 appears prolonged in TC1 and TC3. The temporal Tb response appears to be visible in some clusters, especially TC1, TC4, TC6, and TC7. In comparison to the 50 dB condition, the N2 response appears more distinct in some clusters. Frontocentral N2 responses seem evident in TC4 and TC5, and a later N2 is visible frontally in TC2; rescaled amplitudes also appear somewhat low frontally in TC1 and centrally in TC3. *C* (bottom left). Rescaled amplitude waveforms evoked by 70 dB stimuli. A prolonged fronto-central positivity appears to be visible in TC6. A second positivity following P1 appears evident in TC2, while the P1 seems prolonged in TC1 and TC3. Particularly robust and sustained frontocentral N2 responses are evident in TC1, TC4, and TC5, while the N2 response appears somewhat later in TC2 and TC3. As in the 60 dB condition, there is an early frontal negativity in TC7, followed by little further activity. *D* (bottom right). Rescaled amplitude waveforms evoked by 80 dB stimuli. Patterns are generally similar to those from the 70 dB condition, with some exceptions. The prolonged positivity in TC6 is visible frontally but, compared to the 70 dB condition, is less evident over the central region. In TC5, the N2 appears primarily over the central region; in contrast to the 70 dB condition, there is little evidence of an N2 response to 80 dB stimuli over frontal regions in TC5.

However, in some clusters, central and fronto-central positivities were apparent even after the canonical P1 time window. For example, in TC3, high rescaled amplitudes (per Fig. 3) are clearly apparent, particularly frontally, as late as ~251–275 ms in the 50 dB condition, with similar if lesser prolongations to higher-intensity sounds. In Fig. 4 (see also raw voltage waveforms in Supplementary Figs. A.3–A.6), which depicts rescaled waveforms (TC3 in black) over each of the seven measurement regions (region boundaries given in Fig. 1), these rescaled amplitudes appear to decline gradually from the P1 peak, although in some intensity conditions this gradual decline is interrupted by temporary plateaux approximately coinciding with the temporal Tb negativity.

A slightly different pattern is apparent in TC2, where late high rescaled amplitudes (per Fig. 3) appear to have a more central than frontal distribution. This central positivity appears not to reflect a simple prolongation of the P1 as in TC3, but a separate and distinct response (as can be seen predominantly at mid-central locations in the waveforms from Fig. 4, Supplementary Figs. A.3–A.6; TC2 waveforms are cyan). It also noteworthy that this central positivity in TC2 approximately coincides with the temporal Tb negativity.

T6 may exhibit the most unusual pattern. As in TC3, the P1 response in TC6 appears to continue frontocentrally – especially frontally – long after the P1 time window. However, where the frontal positivity in TC3 does eventually end, the frontal positivity in TC6 continues through to the end of the clustering time window, and it is therefore apparent in all columns of the TC6 section of Fig. 3 from ~51–350 ms. It is also noteworthy that these late frontal positivities appear less intensity dependent than the earlier P1 activation. This pattern is less prominent, but still visible, in raw voltage topographies from Supplementary Fig. A.2.

Finally, TC7 also shows late positive fronto-central voltages that are inconsistent with the canonical morphologies of cortical auditory components in young children. In the rightmost five columns of the TC7 section of Fig. 3, high rescaled amplitudes are apparent in some intensity rows. They are most prominent in the 60 dB condition (second row), where a clear positivity is apparent in raw voltages (Supplementary Fig. A.2).

### Tb Topographies.

Like the P1 component in its canonical window, the temporal Tb negativity generally appears fairly similar across clusters, but there are a few important between-cluster differences. Furthermore, the Tb response is most prominent in responses to higher-intensity stimuli, such as 70 dB or 80 dB sounds. In contrast to the P1, Tb responses to soft 50 dB sounds are difficult to discern in rescaled topographies (Fig. 3) or waveforms (Fig. 4).

Inspection of the temporal region panels (left and right subplots from the middle rows for each intensity) in Fig. 4 reveals that TC6 (green waveform) appears to show weak Tb responses across intensities, but based on statistical results in Appendix C, this may reflect contamination from the sustained frontocentral positivities found in TC6. Instead, the results in Appendix C suggest that raw Tb amplitudes are, in the higher intensity conditions where the Tb response is clearly discernable, particularly strong in TC1 and TC2 compared to other clusters.

Inspection of topographies from Fig. 3 also appears to suggest some substantial between-cluster differences in Tb latencies. For example, in the 80 dB condition (bottom row), the Tb seems to be visible in TC2 from ~151–225 ms, and perhaps as late as ~226–250 ms. In contrast, in clusters such as TC1 and TC5, the Tb appears visible from 126–200 ms. The Tb is visible in TC4 only in the windows from ~126–175 ms. However, inspection of temporal waveforms (Fig. 4) does not appear to suggest substantial Tb latency differences between TC4 and other clusters, perhaps suggesting that the Tb responses in TC4 was subsumed into the early frontocentral N2 negativity that appears in that cluster.

### N2 Topographies.

The N2 response was not consistently evoked in the present study. Although clear N2 responses in the 70 dB and 80 dB conditions (lower two rows of topography plots in Fig. 3) can be seen in some clusters from as early as ~176–200 ms through to ~326–350 ms, the N2 is seldom visible in the 60 dB condition and is never clearly discernable in the 50 dB condition.

Many of topographic differences between the clusters appeared to be related late fronto-central voltages in or shortly prior to the latency range of the canonical N2 negativity. Some of these differences were stark. Particularly clear N2 responses can be seen fronto-centrally in clusters such as TC1 and TC4, and these patterns – highly consistent with canonical patterns – differ sharply from those in some other clusters. As noted previously, TC6 exhibited generally positive frontal voltages throughout the entire time window used in the clustering analysis (~51–350 ms). The only suggestion of any sort of N2 negativity in TC6 was a slight central dip in rescaled voltages in the 80 dB condition (bottom row of TC6 section in Fig. 3), which can be seen in time windows from ~201–350 ms (columns 7–12), but these still coincided with a frontal positivity. Thus, the canonical N2 response appeared absent in TC6.

TC7 offered another unusual case. In TC7, topographies around ~176–200 ms from the 80 dB condition (bottom row in Fig. 3) appear somewhat consistent with a frontal N2 topography. However, given the presence of strong temporal Tb negativities in time windows on either side of the ~176–200 ms period, it seems possible that this “N2” may actually have been part of the Tb response, spread across frontal channels by volume conduction. If so, then TC7 also appears to have lacked a clear N2 response. Fronto-central voltages in TC7 from later time windows seem to have been positive or neutral.

N2 responses varied a great deal across clusters even where they were present. One major between-cluster difference came in the form of latency. N2 responses in TC4 were apparent in topographies very early, around ~176–200 ms in the 60 dB, 70 dB, and 80 dB conditions (bottom three rows in the TC4 section of Fig. 3). Similarly, in TC5, the N2 response was clearly visible in Fig. 3 in the 70 dB condition (third row of TC5 section) from around ~176–200 ms. to ~276–300 ms. In contrast to TC5, the N2 appeared slightly later in TC1 (around ~226–250 ms, and was markedly increased in the 80 dB condition relative to lower intensities, (bottom row of the TC1 section of Fig. 3). The N2 did not appear in TC2 until around ~251–275 ms. In TC3, low/negative central voltages were apparent at that latency, and these had broadened to include frontal channels by ~301–325 ms (the penultimate column of Fig. 3). If the various frontocentral negativities between ~176 and ~350 ms can indeed all be categorized as “N2” responses, then these considerable delays in apparent N2 latencies seem to have permitted other responses to summate over the scalp instead. The central and frontal positivities appearing in TC2 and TC3, respectively – which were previously discussed in the section on topographies of the P1 and other positivities – appeared in the long period before the delayed onset of the N2 in these two clusters.

Clusters also appeared to differ in regard to which sound intensities evoked N2 responses. In TC1, N2 responses were evoked by 70 dB sounds (row 3 of TC1 section of Fig. 3), but these were relatively much weaker than responses to 80 dB sounds (row 4); the N2 response to 60 dB sounds in TC1 was considerably fainter. In contrast, in TC2 and TC4, N2 responses to 60 dB, 70 dB, and 80 dB sounds (rows 2–4 of TC2 and TC4 sections of Fig. 3) appeared much more comparable in rescaled amplitude, although the responses to higher intensities still appeared larger and, at least in the case of TC4, more prolonged. The N2 response in TC5 was especially unusual in its intensity-dependency. Although there was little evidence of an N2 response to high-intensity 80 dB sounds in TC5 (row 4 of TC5 section of Fig. 3), a clear N2 response was elicited by softer 70 dB sounds (row 3).

### Effect of Cluster Membership on Age.

**Fig. 5.**
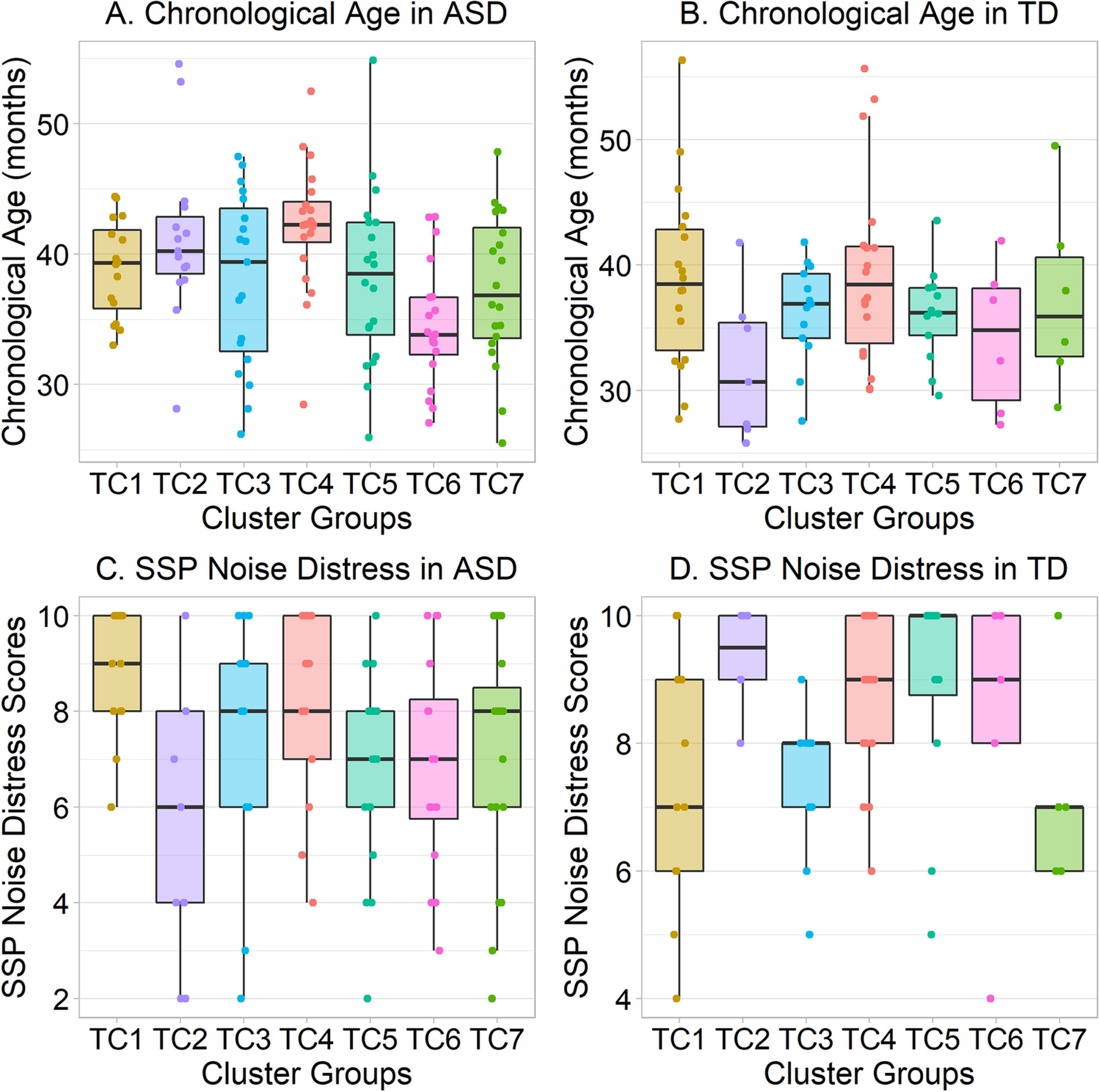
Box-and-whiskers plots displaying the median (central bar) and interquartile range (between lower and upper bars, or hinges) of data, with accompanying jittered data points, showing age and SSP Noise Distress scores of autistic participants across clusters. *A*. Chronological ages of autistic participants in TC1-TC7. Age is significantly lower in TC6 than in TC2 or TC4. *B*. Ages of typically-developing participants in TC1-TC7. No differences attained statistical significance. *C*. SSP Noise Distress scores in autistic participants. Despite an exploratory omnibus effect, no follow-up comparisons attained significance. *D*. SSP Noise Distress scores in typically-developing participants. Despite an exploratory omnibus effect, no follow-up comparisons attained significance. Moreover, some apparent trends are in the opposite direction from ASD effects in panel *C.*

Ages of autistic participants significantly differed across clusters, Kruskal-Wallis *H*(2) = 20.76, *p* = .002 (Fig. 5a). Autistic participants in TC6 were younger than those in TC4, Wilcoxon-Mann-Whitney *p* = .0004, Cliff’s δ = .75, and than those in TC2, Wilcoxon-Mann-Whitney *p* = .02, Cliff’s δ = .63; no other post-hoc effects survived correction. In typically-developing participants, age did not significantly vary across clusters in either solution.

### Effect of Cluster Membership on Sensory, Cognitive, and Adaptive Function Measures.

Although there was no effect of SSP total score, auditory distractibility score, or hyporesponsiveness to speech score (see Table 5), SSP noise distress scores of typically-developing participants significantly varied across clusters, Kruskal-Wallis *H* (6) = 14.60, *p* = .02 (Fig. 5d). Furthermore, the SSP noise distress scores of autistic participants varied across clusters, *H* (6) = 13.16, *p* = .04 (Fig. 5c). However, post-hoc effects did not approach significance in either diagnostic group after correction for multiple comparisons. In addition, apparent trends in either diagnostic group often appeared to reverse direction in the other diagnostic group, which makes interpretation of these data unclear.

There were no significant effects of MSEL DQ or VABS composite score in either diagnostic group or subgroup solution (Table 5).

## Discussion

The present study used hierarchical clustering to describe subgroups of participants in terms of the rescaled scalp topographies of their ERP responses over a broad time window (51–350 ms). Interestingly, most of the variability between clusters observed in the clustering analysis appeared to relate to the topography of responses in the time window canonically associated with the fronto-central N2 response in this age range (i.e., after about ~200 ms; e.g., Shafer et al., 2015, use a 220–388 ms window). Previous research using the same dataset as the present study suggests that this response is attenuated in ASD (Dwyer et al., 2021). The heterogeneous patterns described by the clustering analysis appear to have considerable relevance for the interpretation of this group mean difference. Instead of reflecting a simple reduction in the strength of the N2 negativity in ASD, the mean-level difference between diagnostic groups might at least partly reflect the influence of positive-going responses simultaneously summating over the scalp. The dipole(s) generating these positivities might be relatively stronger in a subset of autistic participants, or the weakness of negative-going responses in these participants might reveal positivities that would otherwise have been masked, or some combination of both factors may be involved.

The present study’s focus on the canonical N2 time window appears to highlight different dimensions of neural variability than those reported on previously by Dwyer et al. (2020), who focused on the topography-independent strengths of Global Field Power responses in the P1 time window across different intensity conditions.

### Responses in Canonical Tb Spatiotemporal Window.

Some differences in Tb response patterns were observed across clusters. For example, the Tb response appeared strong in TC1 and prolonged in TC2. However, between-cluster differences in Tb responses were relatively modest, with all clusters exhibiting, in waveforms, clear Tb responses to at least high-intensity sounds.

### Responses in Canonical N2 Spatiotemporal Window.

However, response patterns in the spatiotemporal window canonically associated with the N2 response differed starkly across clusters. Notably, an N2 response is clearly evoked by 60 dB sounds in TC2, TC4, and TC5, as can be seen in scalp topographies from Fig. 3. However, in TC1, the fronto-central N2 was relatively weak in the 60 dB condition and increased markedly in strength with higher-intensity 70 dB and 80 dB stimuli. Surprisingly, in TC5, there was a clear N2 response to 70 dB stimuli, but there was little evidence of an N2 response to high-intensity 80 dB sounds.

Perhaps even more tellingly, some subgroups exhibit positive-going responses in the time window canonically associated with N2; most strikingly, TC6 does not appear to display a frontocentral N2 response to 70 dB and 80 dB sounds, except perhaps for a slight dip in central amplitudes in response to 80 dB sounds (Figs. 3–5). Instead, in all conditions, TC6 appears to display a sustained frontocentral or frontal positive-going response that begins with the P1 and continues, albeit with reduced amplitudes, until the end of the time window used in the clustering analysis (350 ms). For example, while an early and brief N2 response is visible in TC7 in at least some conditions, this is followed by a positive-going deflection as well as, in the 60 dB condition, positive raw amplitudes (*see* Supplementary Figs. A.2, A.4). Similarly, in TC2, the initial P1 positivity is in all conditions followed, after a negative deflection, by a renewed positivity, particularly over central sites. Although the N2 sometimes (in higher intensity conditions) follows this second positivity in TC2, the positivity does fall partly within the time window used for ANOVA analyses of the canonical N2 at the mean level. The existence of these unusual positive-going responses appears to support our second hypothesis (i.e., that some clusters would exhibit responses with morphologies and topographies not consistent with canonical components).

The presence of these positive-going responses emphasizes the importance of superposition in event-related potential studies. As noted earlier, ERPs are what remains after potentially numerous dipoles from different neural generators summate across the scalp. It seems quite likely that some of the different observed ERP morphologies seen across different subgroups from this analysis might reflect changes in the relative strengths of a number of differently oriented dipoles (associated with either positive or negative voltages at particular scalp sites); such differences in strength could then result in either positive or negative voltage amplitudes over different regions of the scalp.

Moreover, a large number of autistic participants were observed in some of these clusters. For example, there appeared to be many typically-developing participants in TC1 and TC4, and many autistic participants in TC6 and TC7. Although we did not find significant differences in proportions of participants across clusters, this may have reflected the limited power of the chi-square statistic. This makes it difficult to draw clear conclusions regarding the first hypothesis of the present study, which suggested that autistic participants would be more likely to fall into clusters with weaker or absent N2 responses, although some other autistic participants would display responses comparable to typically-developing participants.

As noted earlier, both TC6 and TC7 exhibited ERP response patterns differing from the canonically-expected pattern of negative amplitudes in the N2 time window. A large number of autistic participants also appeared in TC2, with its second central positivity following the P1.

That being said, both typically-developing and autistic participants were present in all clusters, and the lack of a significant chi-square effect highlights the large extent of overlap between diagnostic groups in the distributions of their electrophysiological event-related responses, consistent with the findings in Dwyer et al. (2020) in the same sample as the present study.

### Effects of Cluster Membership on Age and Measured Variables

Interestingly, exploratory analyses comparing clusters on other variables found that autistic participants in TC6 appeared to be unusually young. However, the age effect observed in autism is ambiguous due to the cross-sectional nature of the present study: it is possible, for example, that the younger autistic participants in TC6 might have been able to participate in this study due to being diagnosed earlier than other participants. An early diagnosis might be due to differences in any aspects of behavioural phenotypes or family background not explored in this study, as age of diagnosis in autism is systematically related to other variables (Daniels & Mandell, 2014; Zwaigenbaum et al., 2019).

Exploratory analyses also suggested that the SSP noise distress scores of both autistic and typically-developing participants differed across clusters. However, when the patterns of these significant effects are examined (see Figs. 5C–5D), it is apparent that some clusters characterized by low SSP scores in TD are characterized by high scores in ASD, and vice versa. While one could develop “just-so” stories to explain this apparent interaction, a more prosaic interpretation is simply that the observed effects of SSP noise distress scores reflect Type I error due to the large number of exploratory comparisons run in the present study, and that they should not be further interpreted.

### Limitations

The present study has a number of strengths, including its large and well-characterized sample, the large number of trials collected from each participant, and the use of an intensive data processing pipeline including SOBI independent components analysis to remove putative sources of noise from the final data. However, it is not without limitations.

For example, some age effects were obtained in the present study, and given the cross-sectional nature of the study it is difficult to decisively determine whether these reflect true developmental change or another variable confounded with age. To address this limitation, the authors are currently collecting data in the same experimental paradigm from a new longitudinal sample through the Autism Centers of Excellence-funded Brain Research in Autism Investigating Neurophenotypes (BRAIN) study.

In addition, the present study did not involve collection of hearing acuity measures, due to the difficulty of obtaining reliable estimates of hearing acuity in young children. However, prior research suggests that there is substantial variability in hearing acuity within ASD (Demopolous & Lewine, 2016; Rosenhall et al., 1999). It is unclear how hearing acuity might relate to the individual differences observed in this study.

It should also be noted that the present study’s approach to investigating variability in topography of auditory responses in ASD is only one approach. There are other approaches to topographic analysis, such as TANOVA (see Murray, Brunet, & Michel, 2008); researchers might also choose to attempt to reconstruct sources underlying scalp topographies. Moreover, while the present study investigated intensity-dependency of responses, it does not manipulate dimensions such as frequency or background noise.

Furthermore, the present study only evaluated the stability and replicability of its clustering solution through repeatedly re-sampling and re-clustering subsets of participants (see Appendix B). This approach is limited in the sense that it cannot determine whether the clustering results obtained in the present sample will replicate in other samples. However, the BRAIN study could allow for the replicability of the present study’s results to be investigated.

### Summary

The present study used hierarchical clustering to describe considerable inter-individual variability in the topographies of ERP responses to auditory stimuli in ASD and TD. These results complement and contextualize prior findings of reduced N2 response amplitudes in ASD (see Williams et al., 2020a), including such findings from the dataset used in the present study (Dwyer et al., 2021); on the basis of our results, these findings may to be driven in large part by subgroups with largely absent N2 responses and sometimes, indeed, apparent positive-going responses in the time window associated with the N2. Furthermore, many other autistic participants display responses that cannot be easily distinguished from those of most typically-developing participants, while some typically-developing participants exist in clusters displaying atypical event-related responses. Indeed, the extent of the overlap between diagnostic groups was so considerable that no significant differences in proportions of participants from each group across clusters were observed, despite some visually-apparent trends. This overlap between diagnostic groups, combined with the diverse patterns of neural responses observed across different clusters, emphasizes the existence and importance of inter-individual variability within neurotypes such as ASD and TD. We suggest that clustering analyses similar to those included in the present study may be productively used by other authors to better characterize and understand heterogeneity in their own functional neuroimaging studies of ASD and other neurotypes, thereby placing findings of group mean differences into better context.

## Supplementary Material

1738506_Sup_info

## Figures and Tables

**Table 1.** Characteristics of typically-developing and autistic participants with usable electrophysiological data.

	TD		ASD		Welch’s *p*
	Mean (SD)	Range	Mean (SD)	Range	
Chronological Age (months)	37.09 (6.46)	25.80 – 56.33	38.50 (6.02)	25.50 – 54.87	.12
MSEL Developmental Quotient (DQ)	106.37 (11.58)	79.89 – 128.62	65.25 (20.91)	30.39 – 138.66	<.0001
MSEL Verbal DQ	107.97 (12.70)	81.26 – 149.47	58.90 (26.17)	19.31 – 148.81	<.0001
MSEL Non-Verbal DQ	104.77 (13.88)	71.49 – 129.96	71.60 (18.58)	36.39 – 136.93	<.0001
VABS Adaptive Behaviour Composite	111.22 (12.00)	82.00 – 135.00	75.41 (11.00)	53.00 – 104.00	<.0001

**Table 2.** Mean and standard deviation of number of trials retained in final averages after all data processing was completed by group and condition.

	50 dB	60 dB	70 dB	80 dB
ASD	221.33 (50.29)	212.33 (51.97)	225.64 (49.76)	217.24 (49.77)
TD	240.07 (53.51)	229.49 (54.05)	244.00 (54.35)	234.63 (53.07)
Welch’s *p*	.01	.02	.02	.02

**Table 3.** Counts and percentages of autistic and typically-developing participants, separately, in cluster groups from the seven-cluster solution.

	TC1	TC2	TC3	TC4	TC5	TC6	TC7
ASD	16 (47.06%)	15 (68.18%)	19 (59.38%)	20 (52.63%)	20 (60.61%)	20 (76.92%)	20 (76.92%)
TD	18 (52.94%)	7 (31.82%)	13 (40.63%)	18 (47.37%)	13 (39.39%)	6 (23.08%)	6 (23.08%)

**Table 4.** Counts and percentages of autistic and typically-developing participants, separately, in cluster groups from the seven-cluster solution.

Group	Sex	TC1	TC2	TC3	TC4	TC5	TC6	TC7
ASD	M	13 (81.25%)	13 (86.67%)	16 (84.21%)	17 (85.00%)	16 (80.00%)	17 (85.00%)	18 (90.00%)
	F	3 (18.75%)	2 (13.33%)	3 (15.79%)	3 (15.00%)	4 (20.00%)	3 (15.00%)	2 (10.00%)
TD	M	9 (50.00%)	4 (57.14%)	7 (53.85%)	11 (61.11%)	12 (92.31%)	4 (66.67%)	5 (83.33%)
	F	9 (50.00%)	3 (42.86%)	6 (46.15%)	7 (38.89%)	1 (7.69%)	2 (33.33%)	1 (16.67%)

**Table 5.** Results of Kruskal-Wallis tests comparing autistic and typically-developing participants across clusters TC1-TC7, which were defined on the basis of amplitude and topography of neural response from 51 – 350ms. Both H (Kruskal-Wallis chi-squared) statistics and p-values are reported.

	ASD		TD	
	H(6)	*p*	H(6)	*p*
MSEL DQ	5.64	.46	3.50	.74
VABS Composite	4.18	.65	6.60	.36
SSP Total	5.65	.46	3.31	.77
SSP Auditory Distractibility	11.97	.06	4.98	.55
SSP Hyporesponsiveness to Speech	7.58	.27	3.27	.77
SSP Noise Distress	13.16	.04	14.60	.02

## Data Availability

The data generated in the present study are not publicly available, but are available from the corresponding author on reasonable request.

## Highlights

- Large individual differences in loudness-dependent auditory ERP topographies exist in both autism and typical development
- These individual differences appear particularly clearly in the spatiotemporal window of the N2 response
- These individual differences underlie and can clarify and expand on patterns of group mean differences described in prior research

## Footnotes

1 We use identity-first language (e.g., “autistic person”) in preference to person-first language (e.g., “person with autism”) in deference to prior research indicating that this is preferred by many, albeit not all, autistic people (Bury, Jellet, Spoor, & Hedley, 2020; Kenny et al., 2016), and out of a desire to avoid stigmatizing language (Gernsbacher, 2017). Furthermore, given many autistic people’s aversion to the term “disorder” (Bury et al., 2020), the general unpopularity of the terms “disorder” and “condition” among autistics (Kenny et al., 2016), and the desirability of excluding subjective value judgements from scientific terminology, we have chosen to use the neutral phrase “autism spectrum development” instead.

2 Typically referred to as “attention *deficit* hyperactivity *disorder*.”

## References

[R1] Albrecht R, Suchodoletz W. v., & Uwer R (2000). The development of auditory evoked dipole source activity from childhood to adulthood. Clinical Neurophysiology, 111(12), 2268–2276. 10.1016/S1388-2457(00)00464-8 11090781

[R2] American Psychiatric Association. (2013). Neurodevelopmental disorders. In Diagnostic and statistical manual of mental disorders (5th ed.). 10.1176/appi.books.9780890425596.dsm01

[R3] Andersson S, Posserud MB, & Lundervold AJ (2013). Early and late auditory event-related potentials in cognitively high functioning male adolescents with autism spectrum disorder. Research in Autism Spectrum Disorders, 7(7), 815–823. 10.1016/j.rasd.2013.03.007

[R4] Audras-Torrent L, Miniarikova E, Couty F, Dellapiazza F, Berard M, Michelon C, … Baghdadli A (2021). WISC-V profiles and their correlates in children with autism spectrum disorder without intellectual developmental disorder: Report from the ELENA Cohort. Autism Research, 14(5), 997–1006. 10.1002/aur.2444 33247546

[R5] Ausderau K, Sideris J, Furlong M, Little LM, Bulluck J, & Baranek GT (2014). National survey of sensory features in children with ASD: Factor structure of the sensory experience questionnaire (3.0). Journal of Autism and Developmental Disorders, 44(4), 915–925. 10.1007/s10803-013-1945-1 24097141 PMC3949144

[R6] Belouchrani A, Abed-Meraim K, Cardoso J-F, & Moulines E (1997). A blind source separation technique using second-order statistics. IEEE Transactions on Signal Processing, 45(2), 434–444. 10.1109/78.554307

[R7] Bruneau N, Bonnet-Brilhault F, Gomot M, Adrien JL, & Barthélémy C (2003). Cortical auditory processing and communication in children with autism: Electrophysiological/behavioral relations. International Journal of Psychophysiology, 51(1), 17–25. 10.1016/S0167-8760(03)00149-1 14629919

[R8] Bruneau N, Roux S, Adrien JL, & Barthélémy C (1999). Auditory associative cortex dysfunction in children with autism: Evidence from late auditory evoked potentials (N1 wave-T complex). Clinical Neurophysiology, 110(11), 1927–1934. 10.1016/S1388-2457(99)00149-2 10576489

[R9] Brunet D, Murray MM, & Michel CM (2011). Spatiotemporal analysis of multichannel EEG: CARTOOL. Computational Intelligence and Neuroscience, 2011: 813870. 10.1155/2011/813870 21253358 PMC3022183

[R10] Bury SM, Jellett R, Spoor JR, & Hedley D (2020). “It defines who I am” or “it’s something I have”: What language do [autistic] Australian adults [on the autism spectrum] prefer? Journal of Autism and Developmental Disorders. Advance online publication. 10.1007/s10803-020-04425-3 32112234

[R11] Čeponiene R, Lepistö T, Alku P, Aro H, & Näätänen R (2003). Event-related potential indices of auditory vowel processing in 3-year-old children. Clinical Neurophysiology, 114(4), 652–661. 10.1016/S1388-2457(02)00436-4 12686274

[R12] Čeponiene R, Rinne T, & Näätänen R (2002). Maturation of cortical sound processing as indexed by event-related potentials. Clinical Neurophysiology, 113, 870–882. 10.1016/S1388-2457(02)00078-0 12048046

[R13] Daniels AM, & Mandell DS (2014). Explaining differences in age at autism spectrum disorder diagnosis: A critical review. Autism, 18(5), 583–597. 10.1177/1362361313480277 23787411 PMC4775077

[R14] De Meo-Monteil R, Nordahl CW, Amaral DG, Rogers SJ, Harootonian SK, Martin J, … Saron CD (2019). Differential altered auditory event-related potential responses in young boys on the autism spectrum with and without disproportionate megalencephaly. Autism Research, 12(8), 1236–1250. 10.1002/aur.2137 31157516 PMC7282708

[R15] Demopolous C, & Lewine JD (2016). Audiometric profiles in autism spectrum disorders: Does subclinical hearing loss impact communication? Autism Research, 9(1), 107–120. 10.1002/aur.1495 25962745 PMC4641833

[R16] DiStefano C, Senturk D, & Jeste SS (2019). ERP evidence of semantic processing in children with ASD. Developmental Cognitive Neuroscience, 36: 100640. 10.1016/j.dcn.2019.100640 30974225 PMC6763343

[R17] Donkers FCL, Carlson M, Schipul SE, Belger A, & Baranek GT (2020). Auditory event-related potentials and associations with sensory patterns in children with autism spectrum disorder, developmental delay, and typical development. Autism, 24(5), 1093–1110. 10.1177/1362361319893196 31845589 PMC7297652

[R18] Dwyer P, Wang X, De Meo-Monteil R, Hsieh F, Saron CD, & Rivera SM (2020). Defining clusters of young autistic and typically developing children based on loudness-dependent auditory electrophysiological responses. Molecular Autism, 11: 48. 10.1186/s13229-020-00352-3 32539866 PMC7294610

[R19] Dwyer P, De Meo-Monteil R, Saron CD, & Rivera SM (2021). Effects of age on loudness-dependent auditory ERPs in young autistic and typically-developing children. Neuropsychologia, 156: 107837. 10.1016/j.neuropsychologia.2021.107837 33781752 PMC8102409

[R20] Fletcher-Watson S, & Happé F (2019). Autism: A new introduction to psychological theory and current debate. New York: Routledge.

[R21] Frodl T, Meisenzahl EM, Müller D, Leinsinger G, Juckel G, Hahn K, Möller H-J, & Hegerl U (2001). The effect of the skull on event-related P300. Clinical Neurophysiology, 112(9), 1773–1776. doi:10.1016/S1388-2457(01)00587-9 11514260

[R22] Fushing H, & McAssey MP (2010). Time, temperature, and data cloud geometry. Physical Review, 82: 061110. 10.1103/PhysRevE.82.061110 21230647

[R23] Georgiades S, Szatmari P, Boyle M, Hanna S, Duku E, Zwaigenbaum L, … Thompson A (2013). Investigating phenotypic heterogeneity in children with autism spectrum disorder: A factor mixture modeling approach. Journal of Child Psychology and Psychiatry and Allied Disciplines, 54(2), 206–215. 10.1111/j.1469-7610.2012.02588.x 22862778

[R24] Gernsbacher MA (2017). Editorial perspective: The use of person-first language in scholarly writing may accentuate stigma. Journal of Child Psychology and Psychiatry, 58(7), 859–861. 10.1111/jcpp.12706 28621486 PMC5545113

[R25] Gilley PM, Sharma A, Dorman M, & Martin K (2005). Developmental changes in refractoriness of the cortical auditory evoked potential. Clinical Neurophysiology, 116(3), 648–657. 10.1016/j.clinph.2004.09.009 15721079

[R26] Green SA, Ben-Sasson A, Soto TW, & Carter AS (2012). Anxiety and sensory over-responsivity in toddlers with autism spectrum disorders: Bidirectional effects across time. Journal of Autism & Developmental Disorders, 42(6), 1112–1119. 10.1007/s10803-011-1361-3 21935727 PMC4199633

[R27] Green SA, Hernandez LM, Bowman HC, Bookheimer SY, & Dapretto M (2018). Sensory over-responsivity and social cognition in ASD: Effects of aversive sensory stimuli and attentional modulation on neural responses to social cues. Developmental Cognitive Neuroscience, 29, 127–139. 10.1016/j.dcn.2017.02.005 28284787 PMC5990012

[R28] Grinker RR, Yeargin-Allsopp M, & Boyle C (2011). Culture and autism spectrum disorders: The impact on prevalence and recognition. In Amaral D, Geschwind D, & Dawson G (Eds.), Autism Spectrum Disorders (pp. 62–74). 10.1093/med/9780195371826.003.0008

[R29] Grove R, Begeer S, Scheeren AM, Weiland RF, & Hoekstra RA (2021). Evaluating the latent structure of the non-social domain of autism in autistic adults. Molecular Autism, 12: 22. 10.1186/s13229-020-00401-x 33658064 PMC7931608

[R30] Homan RW, Herman J, & Purdy P (1987). Cerebral location of international 10–20 system electrode placement. Electroencephalography and Clinical Neurophysiology, 66(4), 376–382. 10.1016/0013-4694(87)90206-9 2435517

[R31] Honaker J, King G, & Blackwell M (2011). Amelia II: A program for missing data. Journal of Statistical Software, 45(7), 1–47. 10.18637/jss.v045.i07

[R32] Kenny L, Hattersley C, Molins B, Buckley C, Povey C, & Pellicano E (2016). Which terms should be used to describe autism? Perspectives from the UK autism community. Autism, 20(4), 442–462. 10.1177/1362361315588200 26134030

[R33] Lanka P, Rangaprakash D, Dretsch MN, Katz JS, Denney TS, & Deshpande G (2019). Supervised machine learning for diagnostic classification from large-scale neuroimaging datasets. Brain Imaging and Behavior. Advance online publication. 10.1007/s11682-019-00191-8 PMC7198352 31691160

[R34] Libero LE, Nordahl CW, Li DD, Ferrer E, Rogers SJ, & Amaral DG (2016). Persistence of megalencephaly in a subgroup of young boys with autism spectrum disorder. Autism Research, 9(11), 1169–1182. 10.1002/aur.1643 27273931 PMC5292980

[R35] Lin L-Y, & Huang P-C (2019). Quality of life and its related factors for adults with autism spectrum disorder. Disability and Rehabilitation, 41(8), 896–903. 10.1080/09638288.2017.1414887 29228834

[R36] Lord C, Risi S, Linda L, Cook EH Jr., Leventhal Bennett, L., DiLavore PC, … Rutter M (2000). The Autism Diagnostic Observation Schedule - Generic: A standard measure of social and communication deficits associated with the spectrum of autism. Journal of Autism and Developmental Disorders, 30(3), 205–223. 10.1023/A:1005592401947 11055457

[R37] Lord C, Rutter M, & Le Couteur A (1994). Autism Diagnostic Interview-Revised: A revised version of a diagnostic interview for caregivers of individuals with possible pervasive developmental disorders. Journal of Autism and Developmental Disorders, 24(5), 659–685. 10.1007/BF02172145 7814313

[R38] Luck SJ (2014). An Introduction to the Event-Related Potential Technique (2nd ed.). Cambridge, MA: MIT Press.

[R39] Maris E, & Oostenveld R (2007). Nonparametric statistical testing of EEG- and MEG-data. Journal of Neuroscience Methods, 164(1), 177–190. 10.1016/j.jneumeth.2007.03.024 17517438

[R40] Matsuzaki J, Ku M, Dipiero M, Chiang T, Saby J, Blaskey L, … Roberts TPL (2019). Delayed auditory evoked responses in autism spectrum disorder across the life span. Developmental Neuroscience, 41, 223–233. 10.1159/000504960 32007990 PMC7044064

[R41] McCauley JB, Elias R, & Lord C (2020). Trajectories of co-occurring psychopathology symptoms in autism from late childhood to adulthood. Development and Psychopathology. Advance online publication. 10.1017/S0954579420000826 PMC7655668 32677592

[R42] McIntosh DN, Miller LJ, & Shyu V (1999). Development and validation of the Short Sensory Profile. In Dunn W, Sensory Profile: User’s manual (pp. 59–73). San Antonio, TX: Psychological Corporation.

[R43] Mullen EM (1995). Mullen scales of early learning (AGS ed.). Circle Pines, MN: American Guidance Service.

[R44] Murray MM, Brunet D, & Michel CM (2008). Topographic ERP analyses: A step-by-step tutorial review. Brain Topography, 20(4), 249–264. 10.1007/s10548-008-0054-5 18347966

[R45] Neil L, Olsson NC, & Pellicano E (2016). The relationship between intolerance of uncertainty, sensory sensitivities, and anxiety in autistic and typically developing children. Journal of Autism and Developmental Disorders, 46(6), 1962–1973. 10.1007/s10803-016-2721-9 26864157 PMC4860201

[R46] Nordahl CW, Lange N, Li DD, Barnett LA, Lee A, Buonocore MH, … Amaral DG (2011). Brain enlargement is associated with regression in preschool-age boys with autism spectrum disorders. Proceedings of the National Academy of Sciences, 108(50), 20195–20200. 10.1073/pnas.1107560108 PMC3250128 22123952

[R47] Orekhova EV, Stroganova TA, Prokofyev AO, Nygren G, Gillberg C, & Elam M (2008). Sensory gating in young children with autism: Relation to age, IQ, and EEG gamma oscillations. Neuroscience Letters, 434(2), 218–223. 10.1016/j.neulet.2008.01.066 18313850

[R48] Orekhova EV, Stroganova TA, Prokofiev AO, Nygren G, Gillberg C, & Elam M (2009). The right hemisphere fails to respond to temporal novelty in autism: Evidence from an ERP study. Clinical Neurophysiology, 120(3), 520–529. 10.1016/j.clinph.2008.12.034 19278899

[R49] Parviainen T, Helenius P, & Salmelin R (2019). Children show hemispheric differences in the basic auditory response properties. Human Brain Mapping, 40, 2699–2710. 10.1002/hbm.24553 30779260 PMC6865417

[R50] Perrin F, Pernier J, Bertnard O, Giard MH, & Echallier JF (1987). Mapping of scalp potentials by surface spline interpolation. Electroencephalography and Clinical Neurophysiology, 66(1), 75–81. 10.1016/0013-4694(87)90141-6 2431869

[R51] Ponton C, Eggermont J, Khosla D, Kwong B, & Don M (2002). Maturation of human central auditory system activity: Separating auditory evoked potentials by dipole source modeling. Clinical Neurophysiology, 113, 407–420. 10.1016/S1388-2457(01)00733-7 11897541

[R52] Port RG, Edgar JC, Ku M, Bloy L, Murray R, Blaskey L, … Roberts TPL (2016). Maturation of auditory neural processes in autism spectrum disorder - A longitudinal MEG study. NeuroImage: Clinical, 11, 566–577. 10.1016/j.nicl.2016.03.021 27158589 PMC4844592

[R53] Roberts TPL, Khan SY, Rey M, Monroe JF, Cannon K, Blaskey L, … Edgar JC (2010). MEG detection of delayed auditory evoked responses in autism spectrum disorders: Towards an imaging biomarker for autism. Autism Research, 3(1), 8–18. 10.1002/aur.111 20063319 PMC3099241

[R54] Roberts TPL, Lanza MR, Della J, Qasmieha S, Hines K, Blaskey L, … Berman JI (2013). Maturational differences in thalamocortical white matter microstructure and auditory evoked response latencies in autism spectrum disorders. Brain Research, 1537, 79–85. 10.1016/j.brainres.2013.09.011 24055954 PMC3970268

[R55] Roberts TPL, Matsuzaki J, Blaskey L, Bloy L, Edgar JC, Kim M, … Embick D (2019). Delayed M50/M100 evoked response component latency in minimally verbal/nonverbal children who have autism spectrum disorder. Molecular Autism, 10, 34. 10.1186/s13229-019-0283-3 31428297 PMC6694560

[R56] Rosenhall U, Nordin V, Sandström M, Ahlsén G, & Gillberg C (1999). Autism and hearing loss. Journal of Autism and Developmental Disorders, 29(5), 349–357. 10.1023/A:1023022709710 10587881

[R57] Saggar M, King BG, Zanesco AP, MacLean KA, Aichele SR, Jacobs TL, … Saron CD (2012). Intensive training induces longitudinal changes in meditation state-related EEG oscillatory activity. Frontiers in Human Neuroscience, 6, 256. 10.3389/fnhum.2012.00256 22973218 PMC3437523

[R58] Salmond CH, Vargha-Khadem F, Gadian DG, de Haan M, & Baldeweg T (2007). Heterogeneity in the patterns of neural abnormality in autistic spectrum disorders: Evidence from ERP and MRI. Cortex, 43(6), 686–699. 10.1016/S0010-9452(08)70498-2 17710821

[R59] Shafer VL, Yu YH, & Wagner M (2015). Maturation of cortical auditory evoked potentials (CAEPs) to speech recorded from frontocentral and temporal sites: Three months to eight years of age. International Journal of Psychophysiology, 95(2), 77–93. 10.1016/j.ijpsycho.2014.08.1390 25219893 PMC4346437

[R60] Sharma A, Kraus N, McGee TJ, & Nicol TG (1997). Developmental changes in P1 and N1 central auditory responses elicited by consonant-vowel syllables. Electroencephalography and Clinical Neurophysiology, 104, 540–545. 10.1016/S0168-5597(97)00050-6 9402896

[R61] Sparrow SS, Cichetti DV, & Balla DA (2005). Vineland adaptive behavior scales (2nd ed.). Minneapolis, MN: NCS Pearson.

[R62] Tang AC, Sutherland MT, & McKinney CJ (2005). Validation of SOBI components from high-density EEG. NeuroImage, 25(2), 539–553. 10.1016/j.neuroimage.2004.11.027 15784433

[R63] Tomchek SD, Huebner RA, & Dunn W (2014). Patterns of sensory processing in children with an autism spectrum disorder. Research in Autism Spectrum Disorders, 8(9), 1214–1224. 10.1016/j.rasd.2014.06.006

[R64] Uljarević M, Baranek G, Vivanti G, Hedley D, Hudry K, & Lane A (2017). Heterogeneity of sensory features in autism spectrum disorder: Challenges and perspectives for future research. Autism Research, 10(5), 703–710. 10.1002/aur.1747 28266796

[R65] Whitehouse AJO, & Bishop DVM (2008). Do children with autism “switch off” to speech sounds? An investigation using event-related potentials. Developmental Science, 11(4), 516–524. 10.1111/j.1467-7687.2008.00697.x 18576959

[R66] Williams ZJ, Abdelmessih PG, Key AP, & Woynaroski TG (2020a). Cortical auditory processing of simple stimuli is altered in autism: A meta-analysis of auditory evoked responses. Biological Psychiatry: Cognitive Neuroscience and Neuroimaging. Advance online publication. 10.1016/j.bpsc.2020.09.011 PMC8639293 33229245

[R67] Williams ZJ, He JL, Cascio CJ, & Woynaroski TG (2020b). A review of decreased sound tolerance in autism: Definitions, phenomenology, and potential mechanisms. Neuroscience and Biobehavioral Reviews. Advance online publication. 10.1016/j.neubiorev.2020.11.030 PMC7855558 33285160

[R68] Williams ZJ, Failla MD, Gotham KO, Woynaroski TG, & Cascio C (2018). Psychometric evaluation of the Short Sensory Profile in youth with autism spectrum disorder. Journal of Autism and Developmental Disorders, 48(12), 4231–4249. 10.1007/s10803-018-3678-7 30019274 PMC6219913

[R69] Wunderlich JL, Cone-Wesson BK, & Shepherd R (2006). Maturation of the cortical auditory evoked potential in infants and young children. Hearing Research, 212(1–2), 185–202. 10.1016/j.heares.2005.11.010 16459037

[R70] Yu L, Wang S, Huang D, Wu X, & Zhang Y (2018). Role of inter-trial phase coherence in atypical auditory evoked potentials to speech and nonspeech stimuli in children with autism. Clinical Neurophysiology, 129(7), 1374–1382. 10.1016/j.clinph.2018.04.599 29729592

[R71] Zwaigenbaum L, Duku E, Fombonne E, Szatmari P, Smith IM, Bryson SE, … Bruno R (2019). Developmental functioning and symptom severity influence age of diagnosis in Canadian preschool children with autism. Paediatrics and Child Health, 24(1), e57–e65. 10.1093/pch/pxy076 30906197 PMC6376294

