# Supplementary material for: Using Clustering to Examine Inter-Individual Variability in Topography of Auditory Event-Related Potentials in Autism and Typical Development
Source: Brain Topogr. Author manuscript; Available in PMC 2022 Sep 1. (PMC8436953; doi:10.1007/s10548-021-00863-z)
Supplement: 1738506_Sup_info [file NIHMS1738506-supplement-1738506_Sup_info.docx]

# Online Appendix A.

| 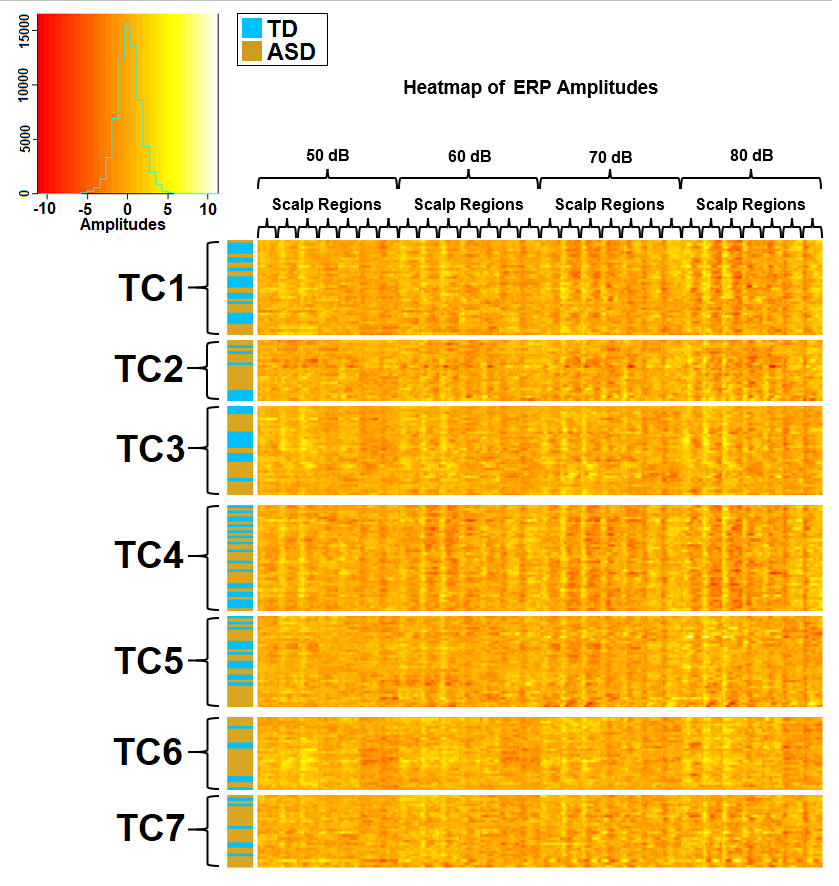  *Supplementary Fig. A.1*. Raw, non-rescaled ERP amplitudes of participants rearranged according to the clustering solution depicted in *Fig. 2* in the main text. The vertical axis shows participants (gold indicates autistic, blue indicates typically-developing). The horizontal axis depicts the four intensity conditions and seven pre-defined scalp regions. Within each scalp region and condition, consecutive 25ms windows are shown from left to right. The scale is provided by a histogram in the upper left corner; the horizontal axis of the histogram shows raw amplitudes. Roughly, brighter (more yellow/white) colours reflect more positive amplitudes, while darker (more red) colours reflect more negative amplitudes. Topographic Clusters 1-7 (TC1-7) are labelled. |
| --- |

| 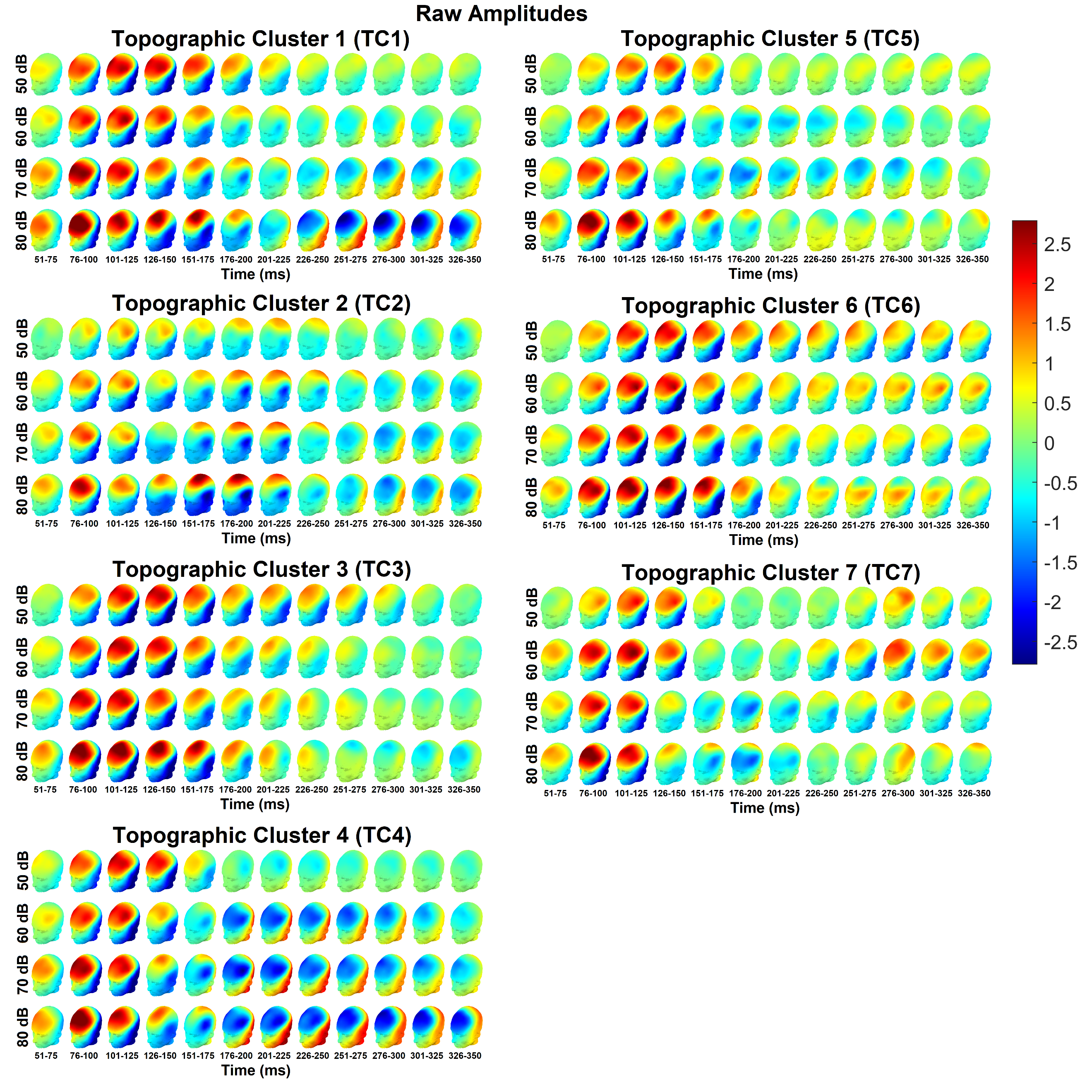 |
| --- |
| *Supplementary Fig. A.2*. Raw ERP amplitudes, averaged across each cluster and sound intensity, collapsed across diagnostic groups, spherically splined from 61 channels and plotted on a head model, in consecutive 25 ms time windows from 51- 350 ms. Compared to rescaled topographies in *Fig. 3*, fronto-central positivities in TC1, TC2, TC3, TC6, and TC7 after ~150 ms are harder to discern. It is also harder to discern the N2 responses in TC2, TC3, and TC5. However, the P1 positivity is similarly visible in raw and rescaled amplitudes, at least near its peaks around the ~76-150 ms time windows. This reflects the manner in which the rescaling procedure enhanced the visibility of some relatively weak responses, while leaving the stronger P1 response largely unaffected. |

| 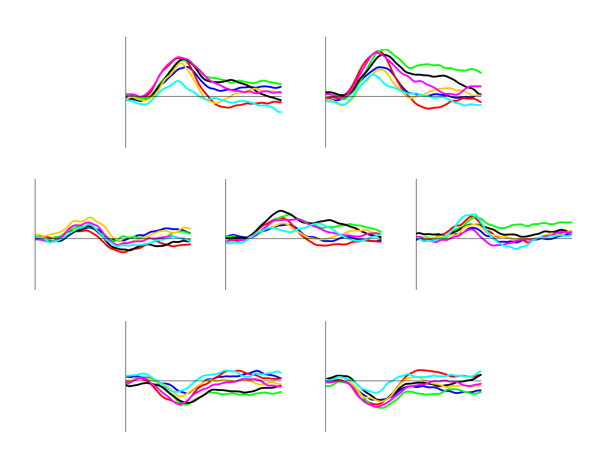  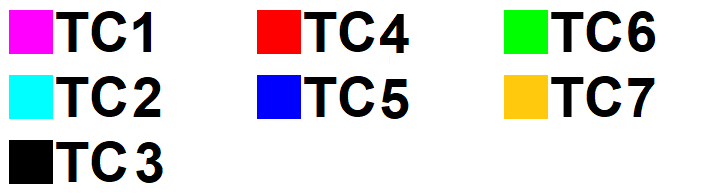 |
| --- |
| *Supplementary Fig. A.3*. ERP raw voltage waveforms evoked by 50 dB sounds over electrodes in each of the seven regions displayed in *Fig. 1* from the main text (left and right frontal subplots at top, central and left and right temporal subplots in middle, left and right posterior subplots at bottom), averaged across each cluster from the seven-cluster solution, collapsed across diagnostic groups. The Y-axis (vertical line on each subplot) ranges from −3.0 to +3.5 μV, while the X-axis ranges from 0 to 350 ms. These raw amplitude waveforms can be compared to the rescaled amplitude waveforms presented in *Fig. 4* from the main text. The relatively larger size of the fronto-central P1 response in the present Fig. in relation to later responses is apparent; as noted in *Fig. 4* of the main text, the rescaling procedure therefore enhances the apparent size of responses from later time points. |

| 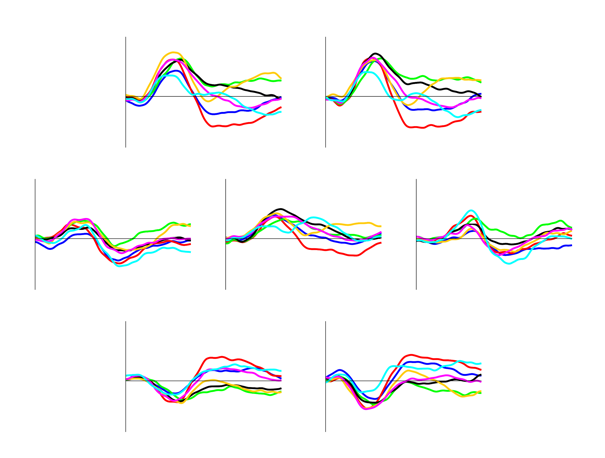  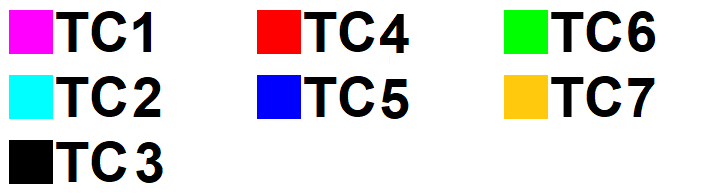 |
| --- |
| *Supplementary Fig. A.4*. ERP raw voltage waveforms evoked by 60 dB sounds over electrodes in each of the seven regions displayed in *Fig. 1* from the main text (left and right frontal subplots at top, central and left and right temporal subplots in middle, left and right posterior subplots at bottom), averaged across each cluster from the seven-cluster solution, collapsed across diagnostic groups. The Y-axis (vertical line on each subplot) ranges from −3.0 to +3.5 μV, while the X-axis ranges from 0 to 350 ms. These raw amplitude waveforms can be compared to the rescaled amplitude waveforms presented in *Fig. 4* from the main text. The relatively larger size of the fronto-central P1 response in the present Fig. in relation to later responses is apparent; as noted in *Fig. 4* of the main text, the rescaling procedure therefore enhances the apparent size of responses from later time points. However, the N2 response in TC4 is also quite large in these raw waveforms; as a result, the rescaling procedure did little to enhance its apparent magnitude. |

| 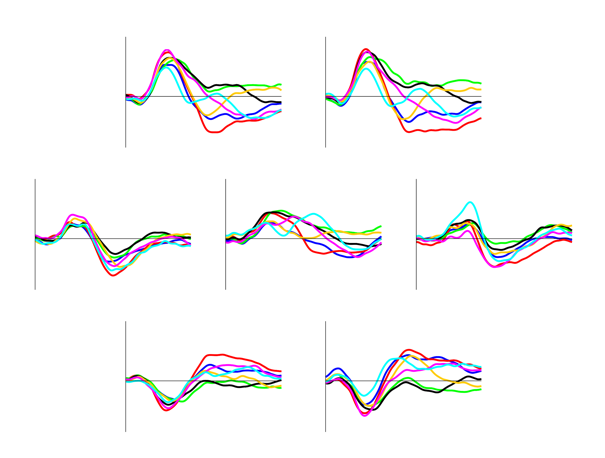  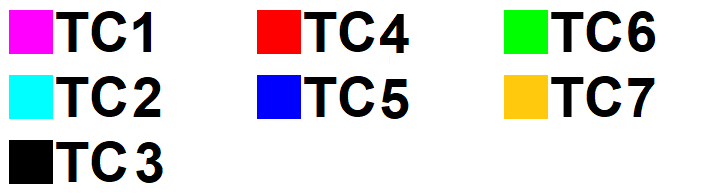 |
| --- |
| *Supplementary Fig. A.5*. ERP raw voltage waveforms evoked by 70 dB sounds over electrodes in each of the seven regions displayed in *Fig. 1* from the main text (left and right frontal subplots at top, central and left and right temporal subplots in middle, left and right posterior subplots at bottom), averaged across each cluster from the seven-cluster solution, collapsed across diagnostic groups. The Y-axis (vertical line on each subplot) ranges from −3.0 to +3.5 μV, while the X-axis ranges from 0 to 350 ms. These raw amplitude waveforms can be compared to the rescaled amplitude waveforms presented in *Fig. 4* from the main text. The relatively larger size of the fronto-central P1 response in the present Fig. in relation to later responses is apparent; as noted in *Fig. 4* of the main text, the rescaling procedure therefore enhances the apparent size of responses from later time points. However, the N2 responses in TC1, TC4, and TC5 are also quite large in these raw waveforms; as a result, the rescaling procedure did little to enhance their apparent magnitude. It should also be noted that the late central positivity in TC2 appears quite large in these raw amplitude waveforms. |

| 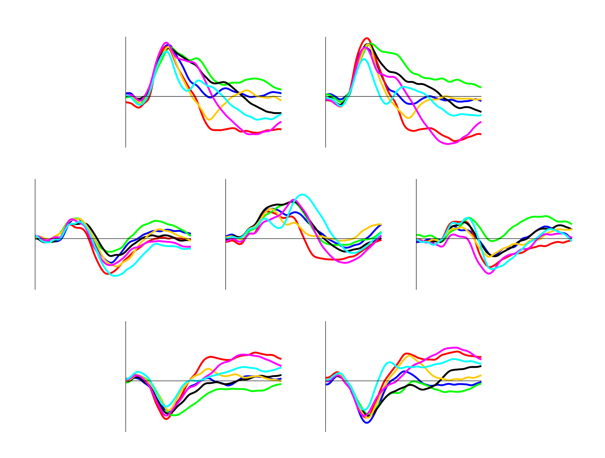  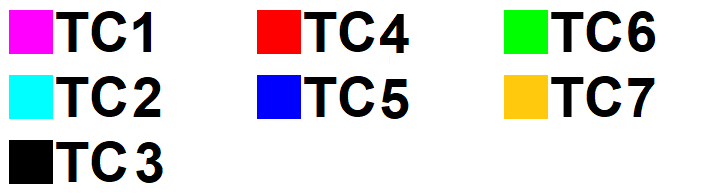 |
| --- |
| *Supplementary Fig. A.6*. ERP raw voltage waveforms evoked by 80 dB sounds over electrodes in each of the seven regions displayed in *Fig. 1* from the main text (left and right frontal subplots at top, central and left and right temporal subplots in middle, left and right posterior subplots at bottom), averaged across each cluster from the seven-cluster solution, collapsed across diagnostic groups. The Y-axis (vertical line on each subplot) ranges from −3.0 to +3.5 μV, while the X-axis ranges from 0 to 350 ms. hese raw amplitude waveforms can be compared to the rescaled amplitude waveforms presented in *Fig. 4* from the main text. The relatively larger size of the fronto-central P1 response in the present Fig. in relation to later responses is apparent; as noted in *Fig. 4* of the main text, the rescaling procedure therefore enhances the apparent size of responses from later time points. However, the N2 responses in TC1 and TC4 are also quite large in these raw waveforms; as a result, the rescaling procedure did little to enhance their apparent magnitude. It should also be noted that the late central positivity in TC2 appears quite large in these raw amplitude waveforms. |

# Online Appendix B.

As an initial exploration of whether participants could be reclassified in the same manner as obtained in the clustering analyses presented in the main text, separately for both the latency and amplitude/topography clustering data, subsamples of 80% of all participants in the present study were resampled 1000 times with replacement. These subsamples were then clustered using Ward’s method (as described in the main text). All solutions with between two and ten clusters were explored. We then calculated the average probabilities that individual participants from each of these clusters would be classified together with other participants that had also been assigned to their cluster in the main analysis, as well as the average probability that participants would be classified together with participants that had been assigned to different clusters in the main analysis. These probabilities were used to aid in selecting the number of clusters to be included in the solutions reported in the main text. The probabilities that correspond to the clustering solution reported in this paper are provided here (Supplementary Table B.1; *Supplementary Fig. B.1*).

Across all of the clusters included in the final solutions described in the main text, averaged probability of being re-classified with participants from this original cluster was greater than averaged probability of being re-classified with participants from other clusters. Furthermore, we observed that clusters TC6 and TC4 from the topographic clustering analysis appeared to be particularly stable, suggesting that these clusters form more stable entities, arguably approaching a more discretely categorical existence, while other clusters from the topographic clustering analysis were more dynamic and fluid, suggesting that these other clusters existed more as constructed groups imposed on dimensional data.

| Supplementary Table B.1. *Averaged probabilities of individual participants (broken down based on assignment to clusters in the seven-subgroup solution from the original topography clustering analysis) being classified together with individual participants that were, or were not, assigned to their cluster in the original topography clustering analysis.* | | | | | | | |
| --- | --- | --- | --- | --- | --- | --- | --- |
| Original Cluster of Participant | TC1 | TC2 | TC3 | TC4 | TC5 | TC6 | TC7 |
| Averaged probability of being re-clustered with participants assigned to the same cluster in the original analysis | .41 | .45 | .44 | .73 | .38 | .78 | .44 |
| Averaged probability of being clustered with participants assigned to other clusters in the original analysis | .13 | .09 | .12 | .08 | .11 | .07 | .10 |

| 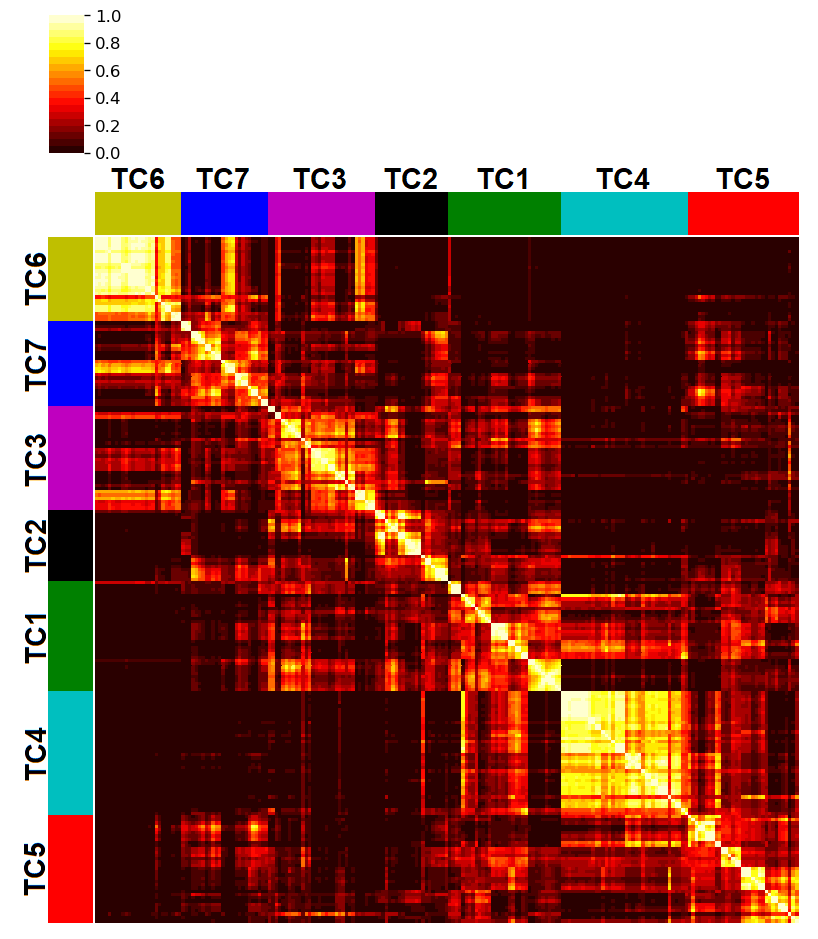 |
| --- |
| *Supplementary Fig. B.1*. Results of a resampling analysis in which data subsets of 80% of participants from topographic clustering analysis were sampled 1000 times with replacement and clustered using Ward’s method to produce seven clusters. Values in the heatmap above are probabilities of each participant (represented as a row/column pair) clustering with every other participant. White and yellow values indicate a higher probability of clustering together. TC4 and TC6 appear remarkably stable, with these clusters being repeatedly recreated in the resampled clustering analyses. Other clusters appear more fluid and unstable, although participants in all clusters are more likely to re-cluster together than apart. |

# Online Appendix C.

To better describe and characterize P1, Tb, and N2 differences between clusters, we performed supplementary analyses examining the amplitudes of these canonical responses and comparing them across clusters.

As a further validation of the rescaling procedure, these analyses were performed using raw amplitudes, to verify that clusters differed not only in rescaled amplitudes but also in raw ERP amplitudes.

## Method

ERP amplitudes from each component were extracted, separately over each hemisphere, as the mean amplitude within a region of interest and a time of interest.

Regions of interest were defined based on manual inspection of the grand-average data and were deliberately selected to be more topographically punctate than the ROIs selected for the clustering analysis, to verify that the between-cluster ERP differences described in the main text can be interpreted in relation to the spatiotemporal windows associated with canonical ERP components (as opposed to spatiotemporally adjacent responses). The selected regions of interest are depicted in *Supplementary Fig. C.1* below.

The P1 time window was defined as ±50ms on either side of the greatest positive peak in the grand-averaged data across both diagnostic groups, yielding the following windows: 73 – 173 ms (50 dB), 60 – 160 ms (60 dB), 45 – 145 ms (70 dB), and 43 – 143 ms (80 dB).

The Tb time window was defined as ±50ms on either side of the greatest negative peak in the grand-averaged data across both diagnostic groups, yielding the following windows: 113 – 213 ms (50 dB), 135 – 235 ms (60 dB), 123 – 223 ms (70 dB), and 113 – 213 ms (80 dB).

As the N2 response peak was poorly defined in the grand-averaged data, at least in softer intensity conditions, the N2 time window was defined as 201 – 350 ms.

Effects of diagnostic group, cluster, hemisphere, and intensity condition on the amplitudes of these ERP responses were statistically examined using four-way mixed ANOVA (Type 3 sums of squares). Further follow-up comparisons were then subsequently conducted where necessary to probe main and interaction effects.

| 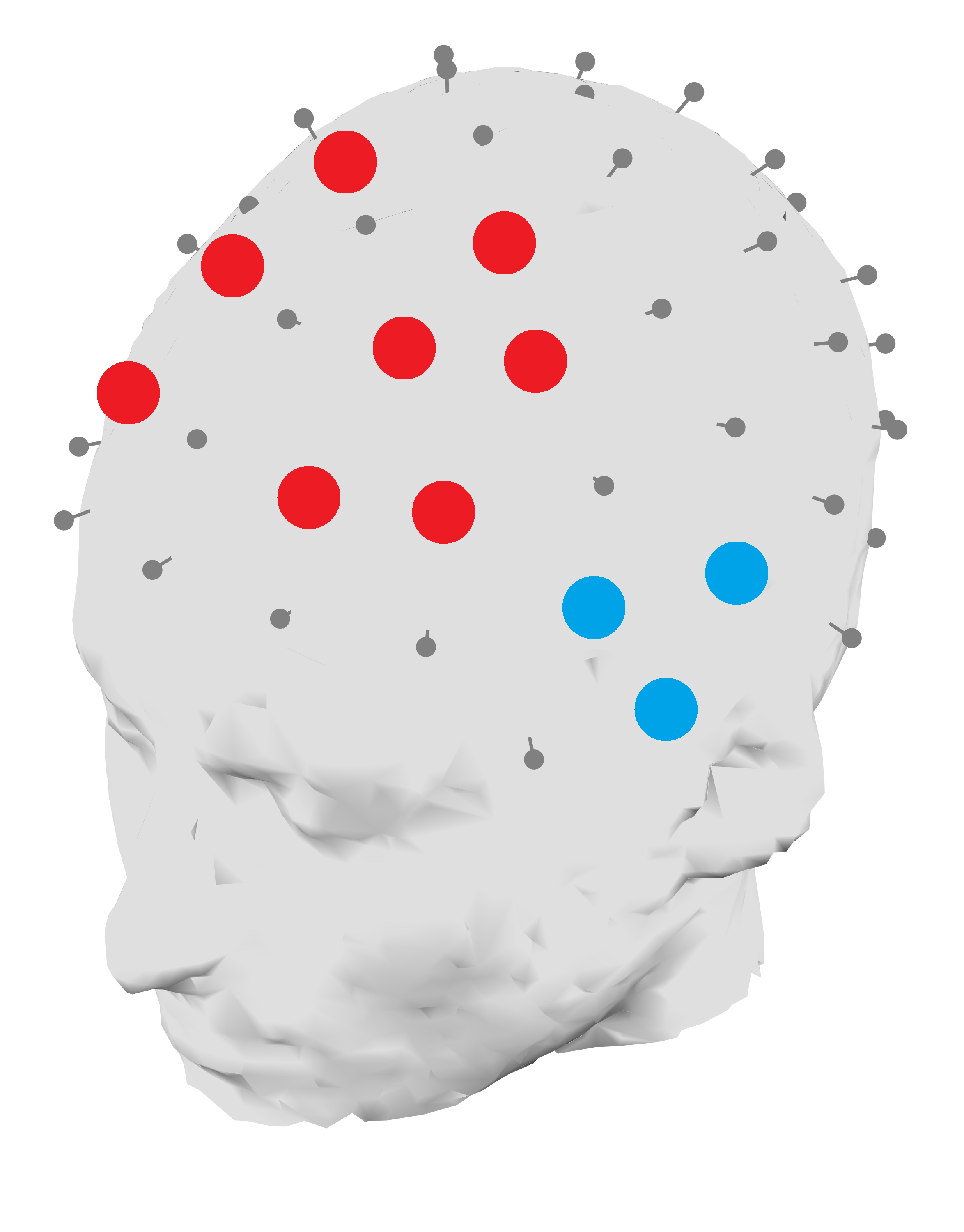  *Supplementary Fig. C.1*. Regions of interest selected for the component-based amplitude analyses. Electrodes of interest for the frontocentral P1 and N2 components are depicted in red. Electrodes of interest for the temporal Tb response are depicted in blue. |
| --- |

## P1 Response.

An omnibus four-way mixed ANOVA (intensity condition x hemisphere x cluster x diagnostic group) found a main effect of cluster on raw P1 amplitudes, *F*(6,197) = 4.52, *p* = .0003, as well as main effect of intensity condition, *F*(3,591) = 6.62, *p* = .0002. There was no main effect of diagnostic group, *F*(1,197) = 1.38, *p* = .24, nor of hemisphere, *F*(1,197) = 0.01, *p* = .92.

Furthermore, there was an interaction between intensity condition and hemisphere, *F*(3,591) = 5.49, *p* = .001, and between cluster and intensity condition, *F*(18,591) = 2.09, *p* = .005. A trending interaction between cluster and hemisphere did not attain significance, *F*(6,197) = 1.89, *p* = .08. No other two-way interactions approached significance.

A three-way interaction of diagnostic group, cluster, and intensity condition was observed, *F*(18,591) = 1.69, *p* = .04, as was a three-way interaction of cluster, hemisphere, and intensity condition, *F*(18,591) = 1.64, *p* < .05. A trending three-way interaction of diagnostic group, hemisphere, and intensity condition did not attain significance, *F*(3,591) = 2.57, *p* = .05. No other three- or four-way interaction effects approached statistical significance.

To probe the significant main effect of intensity, follow-up four-way ANOVAs were conducted comparing each pair of intensity conditions, with other intensity conditions left out. Only main effects of intensity condition are reported, corrected for six multiple comparisons using the Bonferroni-Holm procedure. P1 amplitudes were larger in the 80 dB condition than the 60 dB condition, *F*(1,197) = 10.50, corrected *p* = .008, or the 50 dB condition, *F*(1,197) = 7.19, corrected *p* = .04.

### 50 dB Condition.

In the 50 dB condition, a three-way mixed ANOVA (hemisphere x cluster x diagnostic group) found a main effect of hemisphere on P1 amplitudes, *F*(1,197) = 5.07, *p* = .03, driven by more robustly positive responses over the right hemisphere than the left. There was also a robust main effect of cluster, *F*(6,197) = 4.20, *p* = .0005. There was no main effect of group, *F*(1,197) = 1.39, *p* = .24, nor did any interaction effects approach significance.

Mean P1 amplitudes in each cluster are depicted in Supplementary Table C.1.

| Supplementary Table C.1. *Average P1 amplitudes in each cluster in the 50 dB intensity condition, averaged across hemisphere and diagnostic group, with standard deviations in parentheses.* | | | | | | | |
| --- | --- | --- | --- | --- | --- | --- | --- |
|  | TC1 | TC2 | TC3 | TC4 | TC5 | TC6 | TC7 |
| Amplitude | 1.81 (0.99) | 0.63 (0.98) | 1.71 (1.00) | 1.66 (0.95) | 1.22 (0.83) | 1.79 (1.16) | 1.06 (0.88) |

To further probe the main effect of cluster, Welch’s *t*-tests, corrected for 21 multiple comparisons using the Bonferroni-Holm procedure, were used to compare each pair of clusters, collapsing across hemisphere and diagnostic group. As summarized in Supplementary Table C.2, four effects attained significance after correction. P1 amplitudes were less positive (i.e., weaker) in cluster TC2 than clusters TC1, TC3, TC4, and TC6.

| Supplementary Table C.2. P*-values derived from Welch’s t-tests comparing P1 amplitudes to 50 dB sounds across clusters, collapsing across diagnostic group and hemisphere. Upper values in each cell are uncorrected and lower values are corrected for 21 multiple comparisons using the Bonferroni-Holm procedure.* | | | | | | | |
| --- | --- | --- | --- | --- | --- | --- | --- |
|  | TC1 | TC2 | TC3 | TC4 | TC5 | TC6 | TC7 |
| TC1 |  |  |  |  |  |  |  |
| TC2 | <.0001  .001  ** |  |  |  |  |  |  |
| TC3 | .69 | .0003  .005  ** |  |  |  |  |  |
| TC4 | .52 | .0003  .005  ** | .83 |  |  |  |  |
| TC5 | .01  .16 | .03  .31 | .04  .39 | .04  .36 |  |  |  |
| TC6 | .95 | .0005  .008  ** | .79 | .64 | .04  .39 |  |  |
| TC7 | .003  .05^n.s.^ | .12 | .01  .16 | .01  .16 | .49 | .03  .18 |  |
| For convenience, values are denoted with * if the *p* value is < .05, with ** if *p* < .01, and with *** if *p* < .001. | | | | | | | |

### 60 dB Condition.

In the 60 dB condition, a three-way mixed ANOVA (hemisphere x cluster x diagnostic group) found a main effect of cluster, *F*(6,197) = 3.22, *p* = .005. No other main effects closely approached significance, but an interaction of diagnostic group and hemisphere did attain significance, *F*(1,197) = 4.75, *p* = .03. Trending interactions of diagnostic group and cluster, *F*(6,197) = 1.88, *p* = .09, and cluster and hemisphere, *F*(6,197) = 1.96, *p* = .07, did not attain significance, nor did any other interaction.

To probe the interaction of hemisphere and diagnostic group, we conducted two-way mixed ANOVA examining the effects of cluster and hemisphere within each diagnostic group. In typically-developing participants, there was a main effect of hemisphere, *F*(1,74) = 4.00, *p* < .05, driven by more positive amplitudes over the right hemisphere than the left. There was also a main effect of cluster, *F*(6,74) = 3.20, *p* = .008, and a trending interaction of cluster and hemisphere, *F*(6,74) = 2.22, *p* = .05. In the ASD group, the trending effect of cluster did not attain significance, *F*(6,123) = 1.91, *p* = .09, and there was no effect of hemisphere, *F*(1,123) = 1.02, *p* = .31, nor an interaction of cluster and hemisphere, *F*(6,123) = 1.59, *p* = .16.

To further probe the main effect of cluster, Welch’s *t*-tests, corrected for 21 multiple comparisons using the Bonferroni-Holm procedure, were used to compare each pair of clusters, collapsing across hemisphere and diagnostic group. As summarized in Supplementary Table C.3, no effects attained significance, perhaps reflecting the large correction for multiple comparisons.

| Supplementary Table C.3. P*-values derived from Welch’s t-tests comparing P1 amplitudes to 60 dB sounds across clusters, collapsing across diagnostic group and hemisphere. Upper values in each cell are uncorrected and lower values are corrected for 21 multiple comparisons using the Bonferroni-Holm procedure.* | | | | | | | |
| --- | --- | --- | --- | --- | --- | --- | --- |
|  | TC1 | TC2 | TC3 | TC4 | TC5 | TC6 | TC7 |
| TC1 |  |  |  |  |  |  |  |
| TC2 | .01  .20 |  |  |  |  |  |  |
| TC3 | .69 | .003  .07 |  |  |  |  |  |
| TC4 | .17 | .10 | .06 |  |  |  |  |
| TC5 | .07 | .24 | .02  .38 | .57 |  |  |  |
| TC6 | .62 | .04  .68 | .38 | .46 | .24 |  |  |
| TC7 | .90 | .01  .20 | .81 | .15 | .07 | .56 |  |
| For convenience, values are denoted with * if the *p* value is < .05, with ** if *p* < .01, and with *** if *p* < .001. | | | | | | | |

In addition, paired *t*-tests, corrected for 7 multiple comparisons using the Bonferroni-Holm procedure, were used to compare hemispheres within each cluster. In cluster TC7, raw 60 dB amplitudes were significantly more positive over the left hemisphere than the right, but no other effects achieved significance after correction (Supplementary Table C.4).

| Supplementary Table C.4. *Average P1 amplitudes in each cluster and hemisphere in the 60 dB intensity condition, with standard deviations in parentheses. The final row presents p-values from paired t-tests comparing hemispheres in each cluster; upper values are uncorrected and lower values are corrected for 7 multiple comparisons using the Bonferroni-Holm procedure.* | | | | | | | |
| --- | --- | --- | --- | --- | --- | --- | --- |
|  | TC1 | TC2 | TC3 | TC4 | TC5 | TC6 | TC7 |
| Left | 1.61 (1.05) | 0.80 (1.17) | 1.60 (0.96) | 1.31 (0.98) | 0.98 (0.97) | 1.47 (1.21) | 1.84 (1.04) |
| Right | 1.58 (1.18) | 0.80 (1.29) | 1.77 (1.00) | 1.25 (1.03) | 1.33 (1.11) | 1.45 (0.97) | 1.41 (1.05) |
| p | .82 | .99 | .32 | .67 | .01  .09 | .90 | .006  .04  * |
| For convenience, values are denoted with * if the *p* value is < .05, with ** if *p* < .01, and with *** if *p* < .001 | | | | | | | |

Finally, Welch’s *t*-tests, corrected for 7 multiple comparisons using the Bonferroni-Holm procedure, were used to compare diagnostic groups within each cluster. In cluster TC7, typically-developing participants exhibited more positive P1 amplitudes than autists, but no other effects achieved significance after correction (Supplementary Table C.5).

| Supplementary Table C.4. *Average P1 amplitudes in each cluster and diagnostic group in the 60 dB intensity condition, with standard deviations in parentheses. The final row presents p-values from Welch’s t-tests comparing diagnostic groups in each cluster; upper values are uncorrected and lower values are corrected for 7 multiple comparisons using the Bonferroni-Holm procedure.* | | | | | | | |
| --- | --- | --- | --- | --- | --- | --- | --- |
|  | TC1 | TC2 | TC3 | TC4 | TC5 | TC6 | TC7 |
| ASD | 1.40 (1.18) | 0.71 (1.30) | 1.65 (0.94) | 1.25 (0.87) | 1.05 (0.93) | 1.66 (0.99) | 1.35 (0.90) |
| TD | 1.77 (0.84) | 0.99 (0.65) | 1.73 (1.68) | 1.31 (0.96) | 1.31 (1.04) | 0.81 (0.92) | 2.55 (0.61) |
| p | .30 | .51 | .79 | .84 | .47 | .08 | .003  .02  * |
| For convenience, values are denoted with * if the *p* value is < .05, with ** if *p* < .01, and with *** if *p* < .001 | | | | | | | |

### 70 dB Condition.

In the 70 dB condition, a three-way mixed ANOVA (hemisphere x cluster x diagnostic group) found a main effect of cluster on P1 amplitudes, *F*(6,197) = 3.69, *p* = .002 (Supplementary Table C.6). No other main effects or interactions approached significance.

| Supplementary Table C.6. *Average P1 amplitudes in each cluster in the 70 dB intensity condition, averaged across hemisphere and diagnostic group, with standard deviations in parentheses.* | | | | | | | |
| --- | --- | --- | --- | --- | --- | --- | --- |
|  | TC1 | TC2 | TC3 | TC4 | TC5 | TC6 | TC7 |
| Amplitude | 1.55 (0.84) | 0.70 (0.91) | 1.62 (0.86) | 1.54 (0.80) | 1.03 (0.86) | 1.51 (1.00) | 1.18 (1.07) |

To further probe the main effect of cluster, Welch’s *t*-tests, corrected for 21 multiple comparisons using the Bonferroni-Holm procedure, were used to compare each pair of clusters, collapsing across hemisphere and diagnostic group. As summarized in Supplementary Table C.2, three effects attained significance after correction. P1 amplitudes were less positive (i.e., weaker) in cluster TC2 than clusters TC1, TC3, and TC4.

| Supplementary Table C.7. P*-values derived from Welch’s t-tests comparing P1 amplitudes to 70 dB sounds across clusters, collapsing across diagnostic group and hemisphere. Upper values in each cell are uncorrected and lower values are corrected for 21 multiple comparisons using the Bonferroni-Holm procedure.* | | | | | | | |
| --- | --- | --- | --- | --- | --- | --- | --- |
|  | TC1 | TC2 | TC3 | TC4 | TC5 | TC6 | TC7 |
| TC1 |  |  |  |  |  |  |  |
| TC2 | <.001  .02  * |  |  |  |  |  |  |
| TC3 | .76 | .0006  .01  * |  |  |  |  |  |
| TC4 | .94 | .0008  .02  * | .70 |  |  |  |  |
| TC5 | .01  .22 | .18 | .008  .13 | .01  .20 |  |  |  |
| TC6 | .86 | .005  .13 | .67 | .90 | .06 |  |  |
| TC7 | .15 | .10 | .10 | .15 | .56 | .26 |  |
| For convenience, values are denoted with * if the *p* value is < .05, with ** if *p* < .01, and with *** if *p* < .001. | | | | | | | |

### 80 dB Condition.

In the 80 dB condition, a three-way mixed ANOVA (hemisphere x cluster x diagnostic group) found a main effect of hemisphere on P1 amplitudes, *F*(1,197) = 5.18, *p* = .02, driven by more positive (larger) P1 responses to 80 dB sounds over the left hemisphere than the right hemisphere. There was also a main effect of cluster, *F*(6,197) = 2.76, *p* = .01, and a significant interaction between cluster and hemisphere, *F*(6,197) = 2.81, *p* = .01. No other main effects or interactions approached statistical significance.

To further probe the main effect of cluster, Welch’s *t*-tests, corrected for 21 multiple comparisons using the Bonferroni-Holm procedure, were used to compare each pair of clusters, collapsing across hemisphere and diagnostic group. As summarized in Supplementary Table C.8, one effect attained significance after correction. P1 amplitudes were less positive (i.e., weaker) in cluster TC2 than cluster TC3.

| Supplementary Table C.8. P*-values derived from Welch’s t-tests comparing P1 amplitudes to 80 dB sounds across clusters, collapsing across diagnostic group and hemisphere. Upper values in each cell are uncorrected and lower values are corrected for 21 multiple comparisons using the Bonferroni-Holm procedure.* | | | | | | | |
| --- | --- | --- | --- | --- | --- | --- | --- |
|  | TC1 | TC2 | TC3 | TC4 | TC5 | TC6 | TC7 |
| TC1 |  |  |  |  |  |  |  |
| TC2 | .01  .27 |  |  |  |  |  |  |
| TC3 | .41 | .002  .04  * |  |  |  |  |  |
| TC4 | .65 | .02  .37 | .18 |  |  |  |  |
| TC5 | .95 | .02  .36 | .40 | .72 |  |  |  |
| TC6 | .42 | .003  .06 | .96 | .20 | .40 |  |  |
| TC7 | .21 | .13 | .04  .62 | .34 | .26 | .05^n.s.^ |  |
| For convenience, values are denoted with * if the *p* value is < .05, with ** if *p* < .01, and with *** if *p* < .001. | | | | | | | |

In addition, paired *t*-tests, corrected for 7 multiple comparisons using the Bonferroni-Holm procedure, were used to compare hemispheres within each cluster. No within-cluster interhemispheric differences in amplitudes of P1 responses to 80 dB sounds attained statistical significance after correction (Supplementary Table C.9).

| Supplementary Table C.9. *Average P1 amplitudes in each cluster and hemisphere in the 80 dB intensity condition, with standard deviations in parentheses. The final row presents* p*-values from paired t-tests comparing hemispheres in each cluster; upper values are uncorrected and lower values are corrected for 7 multiple comparisons using the Bonferroni-Holm procedure.* | | | | | | | |
| --- | --- | --- | --- | --- | --- | --- | --- |
|  | TC1 | TC2 | TC3 | TC4 | TC5 | TC6 | TC7 |
| Left | 1.97 (1.02) | 1.26 (1.10) | 2.01 (1.15) | 1.53 (0.97) | 1.85 (1.27) | 1.88 (1.07) | 1.51 (0.82) |
| Right | 1.50 (1.22) | 0.79 (1.13) | 1.87 (0.96) | 1.75 (0.96) | 1.59 (1.19) | 2.02 (1.07) | 1.37 (0.99) |
| p | .009  .06 | .03  .17 | .25 | .16 | .20 | .24 | .39 |

## Tb Response

An omnibus four-way mixed ANOVA (intensity condition x hemisphere x cluster x diagnostic group) found a main effect of hemisphere on raw Tb amplitudes, *F*(1,197) = 27.35, *p* < .0001, driven by more negative amplitudes – a more robust Tb response – over the left hemisphere than the right hemisphere. There was also a main effect of cluster on Tb amplitudes, *F*(6,197) = 4.18, *p* = .0006. A main effect of intensity condition on Tb amplitudes was observed as well, *F*(3,591) = 12.05, *p* < .0001. A strongly-trending main effect of diagnostic group, *F*(1,197) = 3.64, *p* = .06, driven by more negative amplitudes in the TD group, did not attain significance.

Furthermore, there was an interaction between cluster and intensity condition, *F*(18,591) = 2.00, *p* = .008. An interaction between diagnostic group and hemisphere did not attain significance, *F*(1,197) = 3.19, *p* = .08. A trending four-way interaction between diagnostic group, cluster, hemisphere, and intensity condition also fell short of statistical significance, *F*(18,591) = 1.53, *p* = .07. No other interactions approached statistical significance.

### 50 dB Condition.

In the 50 dB condition, a three-way mixed ANOVA (hemisphere x cluster x diagnostic group) found a main effect of diagnostic group on Tb amplitudes, *F*(1,197) = 5.01, *p* = .03, driven by more negative amplitudes (a more robust Tb response) in typically-developing participants. There was also a significant main effect of cluster on raw Tb amplitudes, *F*(6,197) = 2.17, *p* < .05. Finally, the main effect of hemisphere was significant, *F*(1,197) = 16.55, *p* < .0001; this reflected more negative amplitudes (a more robust Tb response) over the left hemisphere than over the right hemisphere. No interactions approached significance.

Mean Tb amplitudes in each cluster are depicted in Supplementary Table C.10.

| Supplementary Table C.10. *Mean Tb amplitudes in each cluster in the 50 dB intensity condition, averaged across hemisphere and diagnostic group, with standard deviations in parentheses.* | | | | | | | |
| --- | --- | --- | --- | --- | --- | --- | --- |
|  | TC1 | TC2 | TC3 | TC4 | TC5 | TC6 | TC7 |
| Amplitude | –0.92 (0.98) | –0.49 (1.05) | –1.02 (0.77) | –0.50 (0.66) | –0.45 (0.72) | –0.67 (0.79) | –0.20 (1.17) |

To further probe the main effect of cluster, Welch’s *t*-tests, corrected for 21 multiple comparisons using the Bonferroni-Holm procedure, were used to compare each pair of clusters, collapsing across hemisphere and diagnostic group. As summarized in Supplementary Table C.11, no effects attained significance, perhaps reflecting the large correction for multiple comparisons.

| Supplementary Table C.11. P*-values derived from Welch’s t-tests comparing Tb amplitudes to 50 dB sounds across clusters, collapsing across diagnostic group and hemisphere. Upper values in each cell are uncorrected and lower values are corrected for 21 multiple comparisons using the Bonferroni-Holm procedure.* | | | | | | | |
| --- | --- | --- | --- | --- | --- | --- | --- |
|  | TC1 | TC2 | TC3 | TC4 | TC5 | TC6 | TC7 |
| TC1 |  |  |  |  |  |  |  |
| TC2 | .12 |  |  |  |  |  |  |
| TC3 | .66 | <.05  .72 |  |  |  |  |  |
| TC4 | .04  .59 | .96 | .004  .07 |  |  |  |  |
| TC5 | .03  .44 | .87 | .003  .06 | .75 |  |  |  |
| TC6 | .28 | .50 | .10 | .36 | .26 |  |  |
| TC7 | .01  .26 | .38 | .004  .07 | .25 | .36 | .10 |  |
| For convenience, values are denoted with * if the *p* value is < .05, with ** if *p* < .01, and with *** if *p* < .001. | | | | | | | |

### 60 dB Condition.

In the 60 dB condition, a three-way mixed ANOVA (hemisphere x cluster x diagnostic group) found a main effect of cluster on Tb amplitudes, *F*(6,197) = 3.89, *p* = .001. In addition, there was a main effect of hemisphere on Tb amplitudes, *F*(1,197) = 13.19, *p* = .0004; this reflected more negative amplitudes (a more robust Tb response) over the left hemisphere than over the right hemisphere. There was no main effect of diagnostic group, *F*(1,197) = 0.72, *p* = .40. No significant interactions were found.

Mean Tb amplitudes in each cluster are depicted in Supplementary Table C.12.

| Supplementary Table C.12. *Mean Tb amplitudes in each cluster in the 60 dB intensity condition, averaged across hemisphere and diagnostic group, with standard deviations in parentheses.* | | | | | | | |
| --- | --- | --- | --- | --- | --- | --- | --- |
|  | TC1 | TC2 | TC3 | TC4 | TC5 | TC6 | TC7 |
| Amplitude | –0.97 (0.94) | –1.72 (1.17) | –0.95 (0.95) | –0.45 (0.99) | –0.85 (1.12) | –0.70 (0.79) | –0.54 (0.79) |

To further probe the main effect of cluster, Welch’s *t*-tests, corrected for 21 multiple comparisons using the Bonferroni-Holm procedure, were used to compare each pair of clusters, collapsing across hemisphere and diagnostic group. As summarized in Supplementary Table C.13, several effects attained significance after correction. Tb amplitudes were more negative (i.e., larger) in cluster TC2 than clusters TC4, TC6, or TC7.

| Supplementary Table C.13. P*-values derived from Welch’s t-tests comparing Tb amplitudes to 60 dB sounds across clusters, collapsing across diagnostic group and hemisphere. Upper values in each cell are uncorrected and lower values are corrected for 21 multiple comparisons using the Bonferroni-Holm procedure.* | | | | | | | |
| --- | --- | --- | --- | --- | --- | --- | --- |
|  | TC1 | TC2 | TC3 | TC4 | TC5 | TC6 | TC7 |
| TC1 |  |  |  |  |  |  |  |
| TC2 | .02  .27 |  |  |  |  |  |  |
| TC3 | .94 | .01 |  |  |  |  |  |
| TC4 | .03  .43 | .0001  .003  ** | .04  .53 |  |  |  |  |
| TC5 | .65 | .009  .16 | .71 | .12 |  |  |  |
| TC6 | .24 | .001  .03  * | .28 | .26 | .55 |  |  |
| TC7 | .06 | .0003  .006  ** | .08 | .71 | .21 | .45 |  |
| For convenience, values are denoted with * if the *p* value is < .05, with ** if *p* < .01, and with *** if *p* < .001. | | | | | | | |

### 70 dB Condition.

In the 70 dB condition, a three-way mixed ANOVA (hemisphere x cluster x diagnostic group) found a main effect of cluster on Tb amplitudes, *F*(6,197) = 2.36, *p* = .03. In addition, there was a main effect of hemisphere on Tb amplitudes, *F*(1,197) = 10.94, *p* = .001; this reflected more negative amplitudes (a more robust Tb response) over the left hemisphere than over the right hemisphere. There was no main effect of diagnostic group, *F*(1,197) = 0.51, *p* = .48. A trending interaction between diagnostic group and hemisphere, *F*(1,197) = 2.97, *p* = .09, did not attain significance, and no other interactions approached significance.

Mean Tb amplitudes in each cluster are reported in Supplementary Table C.14.

| Supplementary Table C.14. *Mean Tb amplitudes in each cluster in the 70 dB intensity condition, averaged across hemisphere and diagnostic group, with standard deviations in parentheses.* | | | | | | | |
| --- | --- | --- | --- | --- | --- | --- | --- |
|  | TC1 | TC2 | TC3 | TC4 | TC5 | TC6 | TC7 |
| Amplitude | –1.44 (0.80) | –1.46 (1.19) | –0.90 (1.05) | –0.91 (0.90) | –0.73 (1.09) | –1.08 (0.79) | –0.54 (1.14) |

To probe the trending interaction of hemisphere and diagnostic group, we conducted two-way mixed ANOVA examining the effects of cluster and hemisphere within each diagnostic group. There was a main effect of hemisphere in ASD, *F*(1,123) = 16.69, *p* < .0001, driven by more negative amplitudes over the left hemisphere than the right. However, no main effect of hemisphere was observed in TD, *F*(1,74) = 1.18, *p* = 28. There was a main effect of cluster in the ASD group, *F*(6,123) = 3.33, *p* = .005, but not in the TD group, *F*(6,74) = 1.19, *p* = .33. Interactions did not approach significance in either group. This implies that Tb amplitudes to 70 dB sounds are, at the group mean level, attenuated in ASD selectively over the right hemisphere.

To further probe the main effect of cluster, Welch’s *t*-tests, corrected for 21 multiple comparisons using the Bonferroni-Holm procedure, were used to compare each pair of clusters, collapsing across hemisphere and diagnostic group. As summarized in Supplementary Table C.15, one effect attained significance after correction. Tb amplitudes were more negative (i.e., larger) in cluster TC1 than cluster TC7.

| Supplementary Table C.15. P*-values derived from Welch’s t-tests comparing Tb amplitudes to 70 dB sounds across clusters, collapsing across diagnostic group and hemisphere. Upper values in each cell are uncorrected and lower values are corrected for 21 multiple comparisons using the Bonferroni-Holm procedure.* | | | | | | | |
| --- | --- | --- | --- | --- | --- | --- | --- |
|  | TC1 | TC2 | TC3 | TC4 | TC5 | TC6 | TC7 |
| TC1 |  |  |  |  |  |  |  |
| TC2 | .94 |  |  |  |  |  |  |
| TC3 | .02  .39 | .08 |  |  |  |  |  |
| TC4 | .01  .19 | .07 | .96 |  |  |  |  |
| TC5 | .004  .07 | .03  .41 | .52 | .45 |  |  |  |
| TC6 | .09 | .21 | .46 | .44 | .16 |  |  |
| TC7 | .001  .03  * | .009  .18 | .23 | .18 | .53 | .06 |  |
| For convenience, values are denoted with * if the *p* value is < .05, with ** if *p* < .01, and with *** if *p* < .001. | | | | | | | |

### 80 dB Condition.

In the 80 dB condition, a three-way mixed ANOVA (hemisphere x cluster x diagnostic group) found a main effect of cluster on Tb amplitudes, *F*(6,197) = 3.74, *p* = .001. In addition, there was a main effect of hemisphere on Tb amplitudes, *F*(1,197) = 13.86, *p* = .0003; this reflected more negative amplitudes (a more robust Tb response) over the left hemisphere than over the right hemisphere. A trending effect of diagnostic group, *F*(1,197) = 3.19, *p* = .08, driven by more negative amplitudes in TD than ASD, did not attain significance. No significant interactions were observed.

Mean Tb amplitudes in each cluster are reported in Supplementary Table C.16.

| Supplementary Table C.16. *Mean Tb amplitudes in each cluster in the 80 dB intensity condition, averaged across hemisphere and diagnostic group, with standard deviations in parentheses.* | | | | | | | |
| --- | --- | --- | --- | --- | --- | --- | --- |
|  | TC1 | TC2 | TC3 | TC4 | TC5 | TC6 | TC7 |
| Amplitude | –1.68 (1.32) | –1.83 (1.13) | –1.18 (0.94) | –0.84 (1.13) | –0.70 (1.44) | –1.00 (0.75) | –0.68 (1.05) |

To further probe the main effect of cluster, Welch’s *t*-tests, corrected for 21 multiple comparisons using the Bonferroni-Holm procedure, were used to compare each pair of clusters, collapsing across hemisphere and diagnostic group. As summarized in Supplementary Table C.17, several effects attained significance after correction. Tb amplitudes were more negative (i.e., larger) in clusters TC1 and TC2 than TC7, and furthermore were more negative in TC2 than TC4 or TC5.

| Supplementary Table C.17. P*-values derived from Welch’s t-tests comparing Tb amplitudes to 80 dB sounds across clusters, collapsing across diagnostic group and hemisphere. Upper values in each cell are uncorrected and lower values are corrected for 21 multiple comparisons using the Bonferroni-Holm procedure.* | | | | | | | |
| --- | --- | --- | --- | --- | --- | --- | --- |
|  | TC1 | TC2 | TC3 | TC4 | TC5 | TC6 | TC7 |
| TC1 |  |  |  |  |  |  |  |
| TC2 | .64 |  |  |  |  |  |  |
| TC3 | .08 | .03  .40 |  |  |  |  |  |
| TC4 | .005  .08 | .002  .04  * | .17 |  |  |  |  |
| TC5 | .005  .09 | .002  .04  * | .12 | .66 |  |  |  |
| TC6 | .01  .20 | .005  .08 | .42 | .50 | .31 |  |  |
| TC7 | .002  .04  * | .0008  .02  * | .07 | .58 | .96 | .22 |  |
| For convenience, values are denoted with * if the *p* value is < .05, with ** if *p* < .01, and with *** if *p* < .001. | | | | | | | |

## N2 Response

An omnibus four-way mixed ANOVA (intensity condition x hemisphere x cluster x diagnostic group) found a main effect of diagnostic group on raw N2 amplitudes, *F*(1,197) = 3.91, *p* < .05, which was driven by more negative amplitudes in typically-developing participants. There was also a main effect of cluster, *F*(6,197) = 37.89, *p* < .0001. Furthermore, there was a main effect of intensity condition, *F*(3,591) = 40.37, *p* < .0001. There was no main effect of hemisphere, *F*(1,197) = 0.02, *p* = .90.

Several interactions also achieved significance. There was an interaction of diagnostic group and hemisphere, *F*(1,197) = 4.42, *p* = .04. There was an interaction of cluster and hemisphere, *F*(6,197) = 3.51, *p* = .003. There was an interaction of cluster and intensity condition, *F*(18,591) = 6.44, *p* < .0001. Finally, there was an interaction of hemisphere and intensity condition, *F*(3,591) = 3.24, *p* = .02.

Follow-up analyses were conducted separately in each intensity condition.

### 50 dB Condition.

In the 50 dB condition, a three-way mixed ANOVA (hemisphere x cluster x diagnostic group) found a main effect of cluster on raw N2 amplitudes, *F*(6,197) = 7.60, *p* < .0001. There was also a main effect of hemisphere, *F*(1,197) = 4.83, *p* = .03, which was driven by more positive N2 amplitudes over the right hemisphere. Finally, there was a significant interaction between hemisphere and cluster, *F*(6,197) = 4.27, *p* = .0005. However, there was no effect of diagnostic group on raw N2 amplitudes in the 50 dB condition, *F*(1,197) = 0.70, *p* = .40, nor was there any interaction involving diagnostic group.

To further probe the effect of cluster, Welch’s *t*-tests, corrected for 21 multiple comparisons using the Bonferroni-Holm procedure, were used to compare each pair of clusters, collapsing across hemisphere and diagnostic group. Numerous between-cluster differences were found, as summarized in Supplementary Table C.18.

| Supplementary Table C.18. P*-values derived from Welch’s t-tests comparing N2 amplitudes to 50 dB sounds across clusters, collapsing across diagnostic group and hemisphere. Upper values in each cell are uncorrected and lower values are corrected for 21 multiple comparisons using the Bonferroni-Holm procedure.* | | | | | | | |
| --- | --- | --- | --- | --- | --- | --- | --- |
|  | TC1 | TC2 | TC3 | TC4 | TC5 | TC6 | TC7 |
| TC1 |  |  |  |  |  |  |  |
| TC2 | .02  .19 |  |  |  |  |  |  |
| TC3 | .12 | .0004  .007  ** |  |  |  |  |  |
| TC4 | .0002  .004  ** | .42 | <.0001  <.0001  *** |  |  |  |  |
| TC5 | .45 | .10 | .03  .28 | .007  .08 |  |  |  |
| TC6 | .003  .04  * | <.0001  .0003  *** | .06 | <.0001  <.0001  *** | .0008  .01  * |  |  |
| TC7 | .46 | .08 | .03  .29 | .005  .06 | .96 | .0007  .01  * |  |
| For convenience, values are denoted with * if the *p* value is < .05, with ** if *p* < .01, and with *** if *p* < .001. | | | | | | | |

In addition, paired *t*-tests, corrected for 7 multiple comparisons using the Bonferroni-Holm procedure, were used to compare hemispheres within each cluster. In cluster TC6, raw 50 dB amplitudes were significantly more positive over the right hemisphere than the left, but no other effects achieved significance after correction (Supplementary Table C.19).

| Supplementary Table C.19. *Average N2 amplitudes in each cluster and hemisphere in the 50 dB intensity condition, with standard deviations in parentheses. The final row presents* p*-values from paired* t*-tests comparing hemispheres in each cluster; upper values are uncorrected and lower values are corrected for 7 multiple comparisons using the Bonferroni-Holm procedure.* | | | | | | | |
| --- | --- | --- | --- | --- | --- | --- | --- |
|  | TC1 | TC2 | TC3 | TC4 | TC5 | TC6 | TC7 |
| Left | 0.36 (0.97) | –0.30 (0.99) | 0.50 (0.81) | –0.47 (0.98) | 0.42 (1.15) | 0.82 (1.10) | 0.20 (1.10) |
| Right | 0.41 (0.99) | –0.15 (1.16) | 0.91 (1.00) | –0.38 (1.01) | 0.01 (1.01) | 1.59 (1.32) | 0.25 (0.71) |
| *p* | .78 | .53 | .01  .07 | .46 | .01  .06 | .0004  .003  ** | .78 |
| For convenience, values are denoted with * if the *p* value is < .05, with ** if *p* < .01, and with *** if *p* < .001 | | | | | | | |

### 60 dB Condition.

In the 60 dB condition, a three-way mixed ANOVA (hemisphere x cluster x diagnostic group) found a main effect of cluster on raw N2 amplitudes, *F*(6,197) = 21.02, *p* < .0001. There was also a nonsignificant trend towards an interaction between diagnostic group and cluster, *F*(6,197) = 2.13, *p* > .05. There were no other significant main effects or interactions.

To further probe the main effect of cluster, Welch’s *t*-tests, corrected for 21 multiple comparisons using the Bonferroni-Holm procedure, were used to compare each pair of clusters, collapsing across hemisphere and diagnostic group. Numerous between-cluster differences were found, as summarized in Supplementary Table C.20.

| Supplementary Table C.20. P*-values derived from Welch’s t-tests comparing N2 amplitudes to 60 dB sounds across clusters, collapsing across diagnostic group and hemisphere. Upper values in each cell are uncorrected and lower values are corrected for 21 multiple comparisons using the Bonferroni-Holm procedure.* | | | | | | | |
| --- | --- | --- | --- | --- | --- | --- | --- |
|  | TC1 | TC2 | TC3 | TC4 | TC5 | TC6 | TC7 |
| TC1 |  |  |  |  |  |  |  |
| TC2 | 0.54 |  |  |  |  |  |  |
| TC3 | .004  .03  * | .002  .02  * |  |  |  |  |  |
| TC4 | <.0001  <.0001  *** | .0002  .002  ** | <.0001  <.0001  *** |  |  |  |  |
| TC5 | .29 | .71 | .0002  .002  ** | .0001  .002  ** |  |  |  |
| TC6 | <.0001  .0003  *** | <.0001  .0002  *** | .05^n.s.^ | <.0001  <.0001  *** | <.0001  <.0001  *** |  |  |
| TC7 | <.0001  <.0001  *** | <.0001  <.0001  *** | .04  .22 | <.0001  <.0001  *** | <.0001  <.0001  *** | .98 |  |
| For convenience, values are denoted with * if the *p* value is < .05, with ** if *p* < .01, and with *** if *p* < .001. | | | | | | | |

In addition, Welch’s *t*-tests were used to compare diagnostic groups within each cluster. No differences between diagnostic groups attained significance in any cluster, even before multiple comparison corrections were applied (Supplementary Table C.21).

| Supplementary Table C.21. *Average N2 amplitudes in each cluster and diagnostic group in the 60 dB intensity condition, collapsed across hemisphere, with standard deviations in parentheses. The final row presents uncorrected* p*-values from Welch’s* t*-tests comparing diagnostic groups in each cluster.* | | | | | | | |
| --- | --- | --- | --- | --- | --- | --- | --- |
|  | TC1 | TC2 | TC3 | TC4 | TC5 | TC6 | TC7 |
| ASD | –0.16 (0.81) | –0.66 (0.82) | 0.54 (0.96) | –1.42 (0.81) | –0.73 (0.86) | 0.99 (1.00) | 0.81 (0.97) |
| TD | –0.43 (0.88) | 0.00 (0.74) | 0.02 (0.69) | –1.29 (0.76) | –0.23 (0.92) | 0.27 (0.84) | 0.92 (0.64) |
| *p* | .35 | .08 | .08 | .61 | .13 | .11 | .74 |

### 70 dB Condition.

In the 70 dB condition, a three-way mixed ANOVA (hemisphere x cluster x diagnostic group) found a main effect of diagnostic group on raw N2 amplitudes, *F*(1,197) = 7.45, *p* = .007, driven by more negative amplitudes in TD than ASD. There was also a main effect of cluster, *F*(6,197) = 18.92, *p* < .0001. Finally, the interaction of diagnostic group and hemisphere achieved significance, *F*(1,197) = 7.76, *p* = .006. No other main effects or interactions attained significance. Mean N2 amplitudes in each cluster are depicted in Supplementary Table C.22.

| Supplementary Table C.22. *Mean N2 amplitudes in each cluster in the 70 dB intensity condition, collapsed across hemisphere and diagnostic group, with standard deviations in parentheses.* | | | | | | | |
| --- | --- | --- | --- | --- | --- | --- | --- |
|  | TC1 | TC2 | TC3 | TC4 | TC5 | TC6 | TC7 |
| Amplitude | –0.98 (1.06) | –0.59 (0.90) | 0.16 (0.95) | –1.52 (0.91) | –0.90 (1.00) | 0.67 (0.95) | 0.21 (0.89) |

To probe the interaction of hemisphere and diagnostic group, we conducted two-way mixed ANOVA examining the effects of cluster and hemisphere within each diagnostic group. There was a trend towards a main effect of hemisphere in ASD, *F*(1,123) = 3.15, *p* = .08, driven by more negative amplitudes over the left hemisphere than the right hemisphere. The main effect of hemisphere attained significance in TD, *F*(1,74) = 4.77, *p* = .03, but in typically-developing participants it was driven by more negative amplitudes over the right hemisphere than the left, in contrast to the ASD pattern.

To further probe the main effect of cluster on N2 amplitudes towards 70 dB complex tones, Welch’s *t*-tests, corrected for 21 multiple comparisons using the Bonferroni-Holm procedure, were used to compare each pair of clusters, collapsing across hemisphere and diagnostic group. Numerous between-cluster differences were found, as summarized in Supplementary Table C.23.

| Supplementary Table C.23. P*-values derived from Welch’s t-tests comparing N2 amplitudes to 70 dB sounds across clusters, collapsing across diagnostic group and hemisphere. Upper values in each cell are uncorrected and lower values are corrected for 21 multiple comparisons using the Bonferroni-Holm procedure.* | | | | | | | |
| --- | --- | --- | --- | --- | --- | --- | --- |
|  | TC1 | TC2 | TC3 | TC4 | TC5 | TC6 | TC7 |
| TC1 |  |  |  |  |  |  |  |
| TC2 | .15 |  |  |  |  |  |  |
| TC3 | <.0001  .0003  *** | .005  .04  * |  |  |  |  |  |
| TC4 | .02  .17 | .0004  .004  ** | <.0001  <.0001  *** |  |  |  |  |
| TC5 | .74 | .25 | <.0001  .0006  *** | .008  .06 |  |  |  |
| TC6 | <.0001  <.0001  *** | <.0001  .0003  *** | .05  .28 | <.0001  <.0001  *** | <.0001  <.0001  *** |  |  |
| TC7 | <.0001  .0002  *** | .003  .03  * | .82 | <.0001  <.0001  *** | <.0001  .0004  *** | .08 |  |
| For convenience, values are denoted with * if the *p* value is < .05, with ** if *p* < .01, and with *** if *p* < .001. | | | | | | | |

### 80 dB Condition.

In the 80 dB condition, a four-way mixed ANOVA (intensity condition x hemisphere x cluster x diagnostic group) found a main effect of cluster on raw N2 amplitudes, *F*(1,197) = 28.62, *p* < .0001. There was also a significant interaction between diagnostic group and hemisphere, *F*(1,197) = 3.99, *p* < .05. No other main effects or interactions attained statistical significance.

Mean N2 amplitudes in each cluster are depicted in Supplementary Table C.24.

| Supplementary Table C.24. *Mean N2 amplitudes in each cluster in the 80 dB intensity condition, collapsed across hemisphere and diagnostic group, with standard deviations in parentheses.* | | | | | | | |
| --- | --- | --- | --- | --- | --- | --- | --- |
|  | TC1 | TC2 | TC3 | TC4 | TC5 | TC6 | TC7 |
| Amplitude | –1.80 (0.89) | –0.70 (0.84) | –0.19 (0.83) | –1.99 (0.97) | –0.04 (0.91) | 0.68 (0.94) | –0.11 (1.36) |

To probe the interaction of hemisphere and diagnostic group, we conducted two-way mixed ANOVA examining the effects of cluster and hemisphere within each diagnostic group. There was a main effect of hemisphere in the TD group, *F*(1,74) = 4.58, *p* = .04, which was driven by more negative amplitudes over the right hemisphere than the left. There was no interaction between hemisphere and cluster in the TD group, *F*(6,74) = 0.49, *p* = .82. In the ASD group, there was no main effect of hemisphere, *F*(1,123) = 0.11, *p* = .74, nor was there an interaction between hemisphere and cluster, *F*(6,123) = 1.49, *p* = .19.

To further probe the main effect of cluster on N2 amplitudes towards 80 dB complex tones, Welch’s *t*-tests, corrected for 21 multiple comparisons using the Bonferroni-Holm procedure, were used to compare each pair of clusters, collapsing across hemisphere and diagnostic group. Numerous between-cluster differences were found, as summarized in Supplementary Table C.25.

| Supplementary Table C.25. P*-values derived from Welch’s t-tests comparing N2 amplitudes to 80 dB sounds across clusters, collapsing across diagnostic group and hemisphere. Upper values in each cell are uncorrected and lower values are corrected for 21 multiple comparisons using the Bonferroni-Holm procedure.* | | | | | | | |
| --- | --- | --- | --- | --- | --- | --- | --- |
|  | TC1 | TC2 | TC3 | TC4 | TC5 | TC6 | TC7 |
| TC1 |  |  |  |  |  |  |  |
| TC2 | <.0001  .0003  *** |  |  |  |  |  |  |
| TC3 | <.0001  <.0001  *** | .03  .19 |  |  |  |  |  |
| TC4 | .38 | <.0001  <.0001  *** | <.0001  <.0001  *** |  |  |  |  |
| TC5 | <.0001  <.0001  *** | .008  .06 | .48 | <.0001  <.0001  *** |  |  |  |
| TC6 | <.0001  <.0001  *** | <.0001  <.0001  *** | .0006  .006  *** | <.0001  <.0001  *** | .005  .05  * |  |  |
| TC7 | <.0001  <.0001  *** | .07 | .79 | <.0001  <.0001  *** | .82 | .02  .14 |  |
| For convenience, values are denoted with * if the *p* value is < .05, with ** if *p* < .01, and with *** if *p* < .001. | | | | | | | |
